# Machine-learning developed an iron, copper, and sulfur-metabolism associated signature predicts lung adenocarcinoma prognosis and therapy response

**DOI:** 10.1186/s12931-024-02839-6

**Published:** 2024-05-14

**Authors:** Liangyu Zhang, Xun Zhang, Maohao Guan, Jianshen Zeng, Fengqiang Yu, Fancai Lai

**Affiliations:** 1 Department of Thoracic Surgery, the First Affiliated Hospital, Fujian Medical University, Fuzhou, 350005 China; 2 Department of Thoracic Surgery, National Regional Medical Center, Binhai Campus of the First Affiliated Hospital, Fujian Medical University, Fuzhou, 350212 China

**Keywords:** Iron/copper/sulfur, LUAD, Immune, Machine-learning, Prognosis

## Abstract

**Background:**

Previous studies have largely neglected the role of sulfur metabolism in LUAD, and no study has combine iron, copper, and sulfur-metabolism associated genes together to create prognostic signatures.

**Methods:**

This study encompasses 1564 LUAD patients, 1249 NSCLC patients, and over 10,000 patients with various cancer types from diverse cohorts. We employed the R package ConsensusClusterPlus to separate patients into different ICSM (Iron, Copper, and Sulfur-Metabolism) subtypes. Various machine-learning methods were utilized to develop the ICSMI. Enrichment analyses were conducted using ClusterProfiler and GSVA, while IOBR quantified immune cell infiltration. GISTIC2.0 and maftools were utilized for CNV and SNV data analysis. The Oncopredict package predicted drug information based on GDSC1. TIDE algorithm and cohorts GSE91061 and IMvigor210 evaluated patient response to immunotherapy. Single-cell data was processed using the Seurat package, AUCell package calculated cells geneset activity scores, and the Scissor algorithm identified ICSMI-associated cells. In vitro experiments was conducted to explore the role of ICSMRGs in LUAD.

**Results:**

Unsupervised clustering identified two distinct ICSM subtypes of LUAD, each with unique clinical characteristics. The ICSMI, comprising 10 genes, was constructed using integrated machine-learning methods. Its prognostic power was validated in 10 independent datasets, revealing that LUAD patients with higher ICSMI levels had poorer prognoses. Furthermore, ICSMI demonstrated superior predictive abilities compared to 102 previously published signatures. A nomogram incorporating ICSMI and clinical features exhibited high predictive performance. ICSMI positively correlated with patients gene mutations, and integrated analysis of bulk and single-cell transcriptome data revealed its association with TME modulators. Cells representing the high-ICSMI phenotype exhibited more malignant features. LUAD patients with high ICSMI levels exhibited sensitivity to chemotherapy and targeted therapy but displayed resistance to immunotherapy. In a comprehensive analysis across various cancers, ICSMI retained significant prognostic value and emerged as a risk factor for the majority of cancer patients.

**Conclusions:**

ICSMI provides critical prognostic insights for LUAD patients, offering valuable insights into the tumor microenvironment and predicting treatment responsiveness.

**Supplementary Information:**

The online version contains supplementary material available at 10.1186/s12931-024-02839-6.

## Introduction

Internationally, lung cancer continues to maintain its untoward status as the primary contributor to cancer-related deaths [1], with lung adenocarcinoma (LUAD) representing the predominant histological subtype [2, 3]. Despite considerable progress in therapeutic approaches for LUAD, the discouraging 5-year overall survival rate remains stagnant at below 20% [4].

Iron, as an indispensable trace element, plays a crucial role in human physiology. A deficiency or excess of iron can significantly impact various biological processes [5]. Notably, cancer cells exhibit an augmented reliance on iron for proliferation, rendering them more vulnerable to iron depletion compared to normal cells. Conversely, elevated iron levels can lead to cytotoxicity via membrane lipid peroxidation, a process referred to as ferroptosis [6, 7]. This iron-dependent form of programmed cell death has been identified as a promising strategy for cancer treatment [8]. While some investigations have hinted at the possible involvement of ferroptosis and iron metabolism in the pathogenesis and suppression of lung cancer, the precise molecular mechanisms underlying these associations remain obscure. Further elucidation of these regulatory factors may provide valuable insights into the development of novel therapeutic strategies for this devastating disease. Copper, an essential micronutrient, exercises a pivotal role in numerous biological processes, including biocompound synthesis, mitochondrial respiration, and antioxidant defense. Disruption of copper homeostasis can lead to oxidative stress and cytotoxicity [9]. Recently, mounting evidence implicates copper in the progression of cancer, particularly in the realms of metastasis, angiogenesis, and proliferation [10]. As a critical cofactor of mitochondrial cytochrome C, copper serves as a vital intermediary in energy metabolism. Consequently, cancer tissues exhibit elevated copper levels relative to healthy tissues, underscoring its integral role in sustaining malignant cellular activity [11]. The versatile element sulfur (S), present in two proteinogenic amino acids – L-cysteine (Cys) and L-methionine (Met) – also comprises a wide array of other biologically significant organic and inorganic small molecules, contributing to the multifaceted nature of this essential nutrient. Sulfur residues participate in the constitution of complex disulfide bond architectures within and intercalated among proteins, thereby influencing crucial biological processes like protein conformation, stability, and catalytic competence [12]. Sulfur-bearing molecules play a multifaceted role in various physiologic processes, including enzyme catalysis, energy transduction, and redox homeostasis. Disruptions in these activities contribute to a wide range of diseases, notably cancer [13]. Recently, Liu et al.'s groundbreaking study revealed a new form of programmed cell death, dubbed disulfidptosis. Characterized by the buildup of intracellular disulfides in glucose-deprived cells with heightened expression of SLC7A11, disulfidptosis differs from both ferroptosis and ferroptosis in its mechanism of execution [14]. Previous studies were predominantly explored genes involved in iron and copper metabolism, while neglecting the potential involvement of genes related to sulfur metabolism. In order to initiate an inquiry into the hitherto unexplored realm of sulfur metabolism in LUAD, and further explore the role of genes related to iron and copper metabolism, we collected genes related to iron, copper and sulfur metabolism, as well as ferroptosis, cuproptosis and disulfidptosis, and conducted extensive research.

This research identified two distinct subtypes of LUAD based on patients' metabolic profiles and developed an Iron, Copper, and Sulfur-Metabolism Index (ICSMI) to predict survival and immune response. Higher ICSMI levels correlated with worse prognosis and reduced immunotherapy effectiveness, suggesting ICSMI's potential as a diagnostic and prognostic tool.

## Methods & materials

### Source data

The inclusion criteria for LUAD patients' data are as follows: (a) diagnosed with histologically confirmed lung adenocarcinoma, excluding other types of lung cancer such as lung squamous cell carcinoma, and so forth, (b) underwent surgical procedures, (c) possessed available overall survival (OS) data, and (d) technical replications were removed if deemed necessary. The datasets TCGA-LUAD, GSE72094, GSE68465, and GSE31210 fulfilled these criteria. For other cancer patients' data, the inclusion criteria are as follows: (a) underlying surgical procedures, (b) probable available overall survival (OS) data, and (c) technical replicates were removed if necessary. Data on LUAD patients' clinical information, transcriptomic data, as well as CNV and SNV data were downloaded from the TCGA database (https://portal.gdc.cancer.gov/) [15]. The TCGA-Pancancer dataset contains data on more than 10,000 patients with 33 different cancers, also obtained from the TCGA website. The SNV data was processed by the R package Maftools, and the CNV data was analyzed using GISTIC2.0 [16]. Nine GEO datasets for lung cancer patients were obtained from the GEO database (https://www.ncbi.nlm.nih.gov/geo/)[17], namely GSE68465, GSE72094, GSE31210, GSE37745, GSE41271, GSE3141, GSE30219, GSE42127, and GSE81089. GSE31210, GSE72094, and GSE68465 are cohorts exclusively comprise of LUAD patients; while GSE30219, GSE37745, GSE41271, GSE42127, GSE3141, and GSE81089 are cohorts consisted of patients with variety of NSCLC type. Besides, GSE91061, a dataset includes information for cancer patients receiving immunotherapy, and GSE34228, which contains LUAD cell lines’ sensitivity to gefitinib, were also downloaded from GEO. Additionally, we gathered transcriptomic and clinical data from cancer patients who underwent anti-PD-L1 treatment within the IMvigor210 cohort. This information was sourced from the following reference: http://research-pub.gene.com/IMvigor210CoreBiologies [18]. The single-cell RNA-sequencing dataset GSE127465 was acquired from the TISCH database [19] and processed in accordance with previously outlined procedures [20]. The genes associated with iron and copper metabolism were compiled from previously published research [21–24]. From the MsigDB database [25], we obtained genes associated with sulfur matabolism from GO_SULFUR_COMPOUND_METABOLIC_PROCESS, GO_SULFUR_COMPOUND_BIOSYNTHETIC_PROCESS, and KEGG_SULFUR_METABOLISM genesets. Considering that iron is involved in ferroptosis, copper is involved in cuproptosis, and sulfur is involved in disulfidptosis, we also included genes associated with these three cell death modes for research [14]. As a result, we identified 839 Iron, Copper, and Sulfur Metabolism Related Genes (ICSMRGs, Table S1). LUAD patients' TIDE scores, which predict ICB response, were calculated on the TIDE website (http://tide.dfci.harvard.edu) [26].

### Consensus clustering

Following the application of a powerful clustering method using the ConsensusClusterPlus package [27], we effectively identified two subgroups within the LUAD patient population based on the genetic characteristics of 24 prognostic ICSMIGs. LUAD patients in these two subgroups displayed significant differences in clinical and prognostic attributes across four separate cohorts.

### Construction of the iron, copper and sulfur-metabolism index

Leveraging ten machine learning algorithms (GBM, RSF, SuperPC, Survival-SVM, Lasso, stepwise Cox, Ridge, Enet, CoxBoost, and plsRcox), we developed an integrative Iron, Copper, and Sulfur-Metabolism Index (ICSMI) via the CoxBoost + GBM combination. After conducting a thorough evaluation of 114 varied permutations, we opted for this selection, which mirrored our previous approach [20]. The detailed introduce of each algorithm and the specific implementations of various combinations were illustrated in Supplementary Methods. To validate the predictive efficacy of ICSMI, we calculated the area under the receiver operating characteristic curve (AUC) utilizing the timeROC package. Moreover, we performed Cox regression analysis using the survival package in R to affirm the independent prognostic significance of ICSMI. Additionally, we retrospectively compiled 102 signatures established by prior researchers and contrasted ICSMI's hazard ratio (HR) value and C-index with these markers.

### Batch effect mitigation and integration: creating unified meta cohorts

We employed the "combat" function from the sva package to mitigate batch effects present in the TCGA, GSE72094, GSE68465, and GSE31210 datasets, integrating them into a unified dataset termed Meta. Principal component analysis (PCA) highlighted notable batch effects across the four datasets before applying batch effect removal (Supplementary Fig. 1C), which were successfully alleviated post-integration (Supplementary Fig. 1D).

### Functional enrichment analysis

To uncover the biological pathways linked with ICSMRGs and ICSMI, we conducted enrichment analyses including GO, KEGG, and GSEA using the R package ClusterProfiler [28]. Moreover, we utilized the GSVA package [29] to perform GSVA analysis, further uncovering the potential mechanisms involved.

### Quantifying patients' immune infiltration level

Seven different algorithms were used to assess LUAD patients’ immune cell infiltration in the TCGA dataset. These algorithms included quantTIseq, TIMER, EPIC, MCP-counter, ESTIMATE, and xCell were implemented using the R package 'IOBR' [30]. Besides, ssGSEA was performed by GSVA package. Additionally, the correlation of immune-related molecules’ expression and ICSMI were analyzed.

### The scissor algorithm

To identify the particular cell populations responsible for the noticed variances in ICSMI status, we utilized the Scissor algorithm available in the 'Scissor' package [31]. By harnessing both bulk data and phenotypic information, this methodology facilitates the automated selection of cell subpopulations from single-cell datasets that predominantly contribute to divergent phenotypes. In our study, we compared high-ICSMI patients and low-ICSMI patients within the TCGA cohort, treating these groups as distinct phenotypes. Utilizing transcriptomics data of the high- and low-ICSMI phenotypes across all patients, we applied the 'Scissor' function to associate each cell in the GSE127465 dataset with its corresponding phenotype. By designating Scissor + cells as those most relevant to the high-ICSMI phenotype and Scissor- cells as those most pertinent to the low-ICSMI phenotype, we identified differentially expressed genes (DEGs) between these cell populations using Seurat's 'FindAllMarkers' function. Specifically, genes displaying a fold change exceeding |log2 (fold change)|> 0.25 with an adjusted *p*-value (Padj) below 0.05 were considered significant DEGs.

### Analysis of cell–cell communication in the TME

Using the 'CellChat' package [32], we explored intercellular interaction within the TME, identifying various ligand–receptor pairs that facilitate cross-talk between different cell types.

### Finding potential drugs targeting ICSMI

By combining the data from the GDSC1 database (https://www.cancerrxgene.org/) [33] and the 'oncoPredict' package [34], we evaluated the susceptibility of LUAD samples to diverse therapeutics, as reflected by their IC50 values. The IC50 value represents the concentration at which a drug achieves 50% inhibition of biological processes, typically measured in in vitro experiments. In cancer research, it is commonly used to assess the degree of inhibition a drug exerts on tumor cells. A lower IC50 value indicates greater sensitivity, meaning the drug achieves a significant inhibitory effect at a lower concentration. This enabled us to identify potential targets for personalized medicine strategies.

### Cell culture and transfection

The BEAS-2B normal bronchial epithelial cell line and three LUAD cell lines (A549, PC9, H1975) were obtained from the Cell Bank of the Chinese Academy of Sciences. These cells were cultured at 37 °C with 5% CO2 in DMEM medium (Bioscience, China) supplemented with 10% FBS (Gibco, USA). Small interfering RNA (siRNA), specifically si-GCDH and its corresponding negative control, si-NC, were procured from Hanheng Biology (Shanghai, China). Utilizing Lipofectamine 3000 (Invitrogen, Carlsbad, CA, USA), transfection of siRNA into cells was conducted according to the manufacturer's instructions.

### qRT-PCR

Total RNA extraction was performed using an RNA extraction kit (Vazyme, China) following the manufacturer's instructions. The extracted RNA was reverse transcribed into cDNA using the All-in-One First-Strand Synthesis MasterMix kit (iScience, China). Subsequently, triplicate aliquots of each cDNA sample were prepared using the Taq SYBR® Green qPCR Premix (iScience, China). In this study, the internal reference gene utilized was β-Actin, and the primers for the five ICSMRGs and β-Actin are listed in Table S5.

### Western blotting

Total protein extraction from cells was achieved using RIPA lysis buffer (Meilun Biotechnology, China). Protein concentration was determined using a bicinchoninic acid protein assay kit (#23,227, Thermo Fisher Scientific, Waltham, USA). Denatured proteins were separated by 10% SDS-PAGE and transferred onto nitrocellulose membranes (Millipore in Bedford, USA). Following a 2-h blocking step with 5% skimmed dry milk, the membranes were incubated overnight at 4 °C with primary antibodies, namely anti-GCDH (1:1000, Immunoway), and anti-β-Actin (1:1000, Immunoway), followed by incubation with horseradish peroxidase labeled secondary antibodies (ab7090, 1:5000; Abcam). β-Actin served as a normalization control for the expression of target proteins.

### Wound healing assays

A549 and PC9 LUAD cells, post-transfection, were plated at a density of 10^5^ cells per well in 6-well plates. After 24 h of incubation, when cells reached approximately 80% confluence, a 10-μl pipette tip was employed to create uniform scratches on the cell monolayers. Subsequently, detached cells were gently washed away using PBS, and the bottom of the dish was marked for reference. The wound area of each sample was documented at both 0-h and 24-h time points, with quantitative analysis performed using ImageJ software.

### Transwell assays

Invasion and migration assays were conducted using Transwell chambers (Scipu002872; Corning Inc., Corning, USA). For the cell invasion assay, the Transwell chamber inserts were precoated with 10 μg Matrigel before the experiment. In the upper chamber, 10,000 cells with 200 μl FBS-free DMEM medium were seeded, while 600 μl of culture medium containing 10% FBS was added to the lower chamber. Following a 24-h incubation period at 37 °C, the cells remaining attached to the membrane were fixed with polyformaldehyde and subsequently stained with hematoxylin. Finally, the cells in the lower chamber were photographed under a high-powered microscope.

### Statistic analysis

All statistical analyses were conducted using R (version 4.1.1). Group disparities were assessed using either the Wilcoxon test or t-test, while correlations were examined through Pearson or Spearman correlation coefficients. The log-rank test was utilized for overall survival comparisons. To assess the prognostic impact of ICSMI and clinicopathological factors, multivariate Cox regression analysis was performed. Comparison of multiple signatures' C-Index was carried out using the CompareC package. For *P* values, 'Ns' denotes *P *≥ 0.05, '' signifies *P* < 0.05, '' indicates *P* < 0.01, and '' represents *P* < 0.001.

## Results

### Identification of 24 hub ICSMRGs

**Fig. 1:**
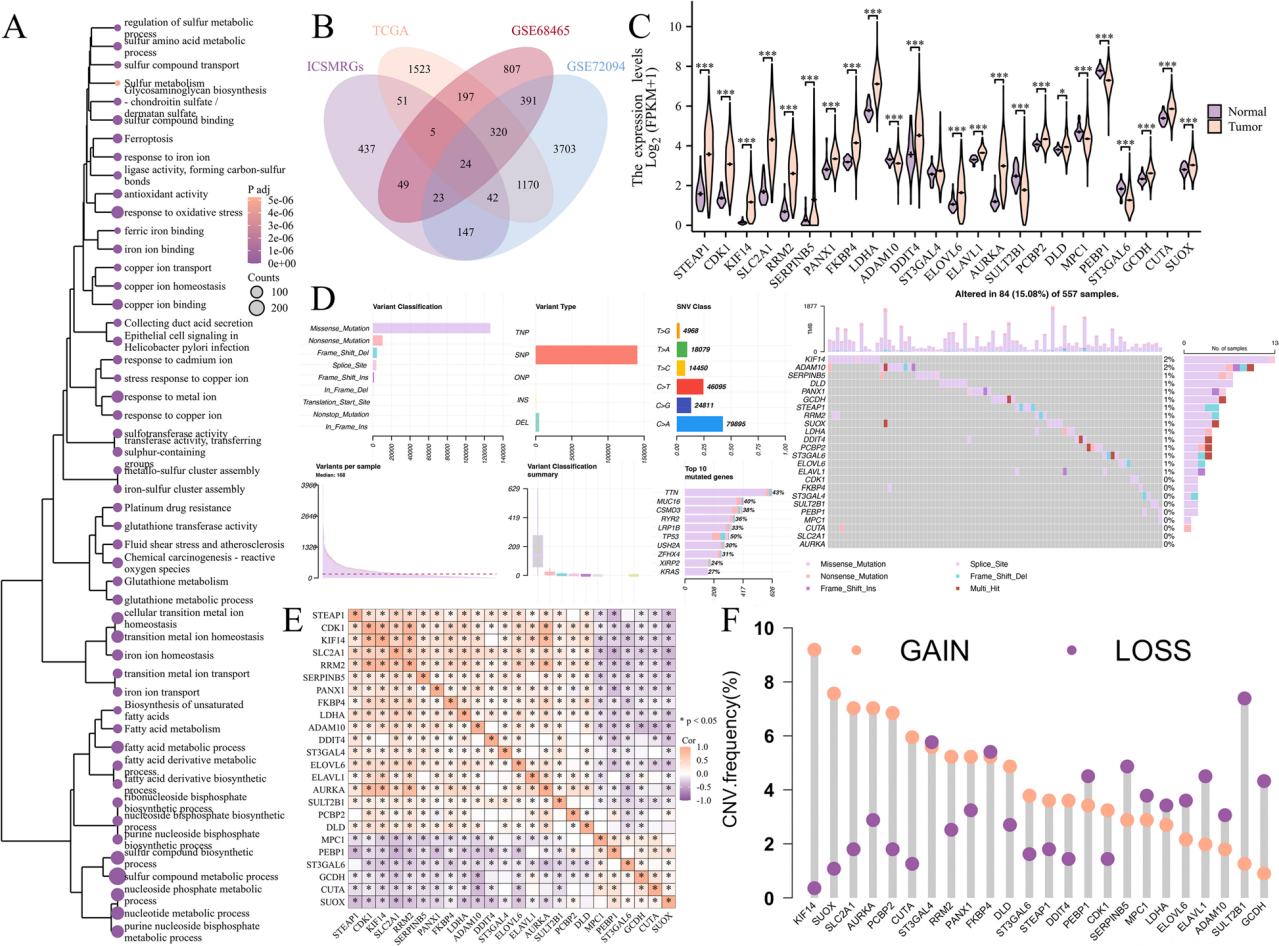
Identification of 24 key ICSMRGs. **A** GO and KEGG analyses demonstrated that these 839 genes we collected were primarily involved in processes related to iron, copper, and sulfur metabolism. **B** 24 ICSMRGs exhibited consensus prognostic value across three cohorts. **C** Differential expression analysis revealed most ICSMRGs exhibiting altered expression patterns between normal and LUAD tissues. **D**, **F** These ICSMRGs harbored both SNVs (**D**) and CNVs (**F**), indicating their potential role in driving tumorigenesis. **E** Correlation heatmap illustrated the interconnected relationships among the 24 genes, highlighting their complex regulatory interactions

After acquiring 839 Iron, Copper, and Sulfur-Metabolic Related Genes (ICSMRGs), we conducted enrichment analysis on them, and divulged that these genes participate in biological processes pertinent to iron, copper, and sulfur metabolism, including 'response to copper ion', 'ferroptosis', 'iron ion homeostasis', 'copper ion homeostasis', 'sulfur compound metabolic process', and 'sulfur amino acid metabolic process'(Fig. 1A). This means that we have successfully identified a series of genes highly correlated with iron, copper, and sulfur metabolism. To identify genes with reliable prognostic value, we employed univariate Cox regression analysis in the TCGA, GSE72094, and GSE68465 cohorts based on their largest sample size, and setting the threshold at 0.05. Subsequently, we intersected the prognostic genes from the three cohorts with 839 ICSMRGs, resulting in 24 ICSMRGs with consistent prognostic significance (Fig. 1B, Table S2). Then we conducted a comparative analysis of the differential expression patterns of these 24 genes between normal and tumor tissues in TCGA, revealing that the majority of them exhibited significant differential expression (Fig. 1C). In TCGA cohort, 22 ICSMRGs were found to harbor varying levels of mutational activity, with the majority comprising missense mutations; the overall mutation frequency was recorded at 15.08% (Fig. 1D). Notably, KIF14 displayed the highest mutation frequency among these ICSMRGs. Concomitantly, we observed diverse degrees of DNA copy number variation in these ICSMRGs, with the majority exhibiting variable CNVs; KIF14 also displayed highset CNV amplifications (Fig. 1F). Correlation analyses disclosed intricate relationship amidst the 24 ICSMRGs, encompassing both positive and adverse associations (Fig. 1E).

### Consensus clustering classifying LUAD patients into two Clusters

**Fig. 2:**
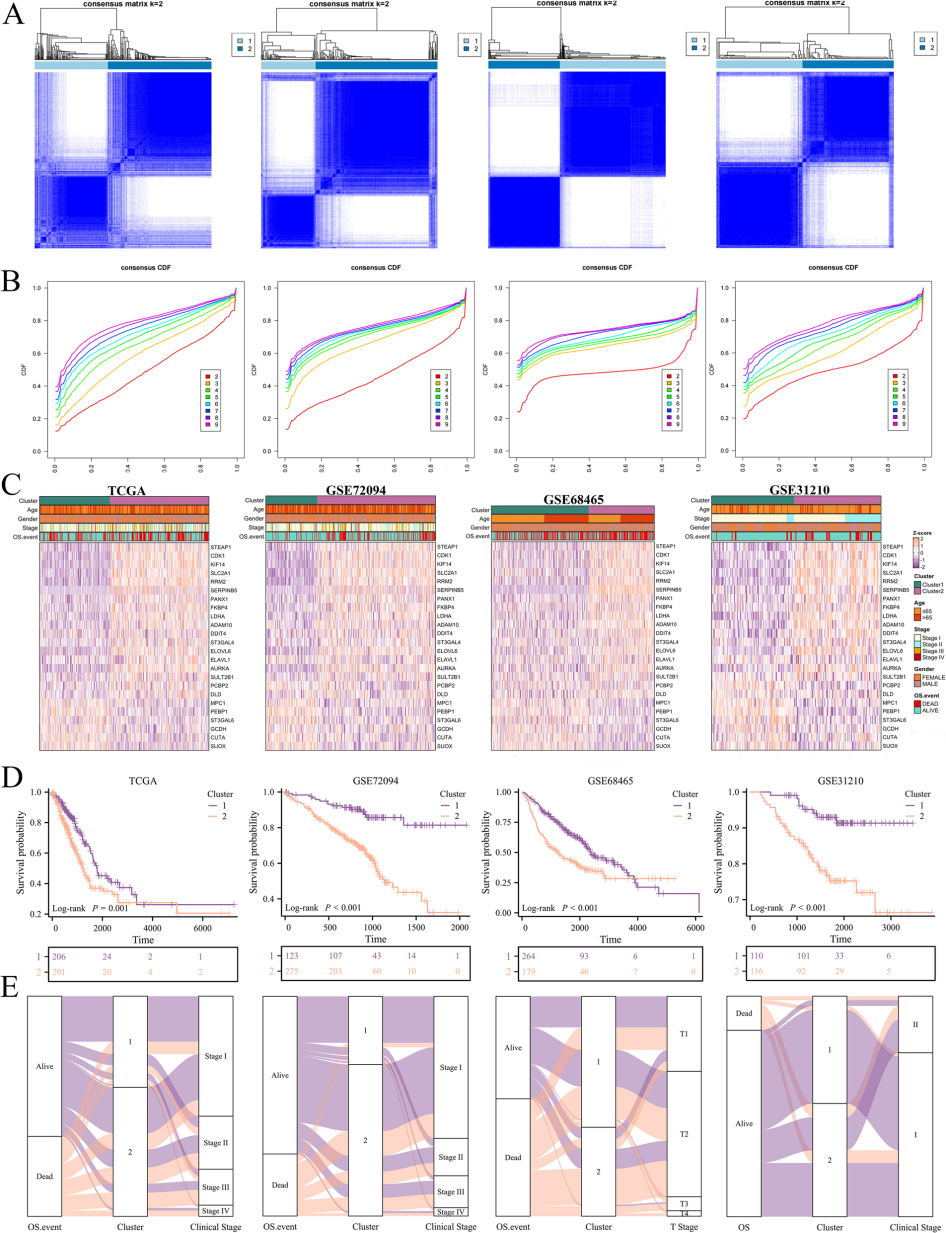
Identification of Two Distinct ICSM Clusters through Consensus Clustering. **A** LUAD patients were grouped into two molecular clusters (k = 2) based on 24 ICSMRGs. **B** The empirical cumulative distribution function plot depicts the consensus distribution for each k value. **C** A heatmap illustrates the expression profiles of the 24 ICSMRGs across the two clusters. **D** Survival analysis reveals significant differences in prognosis between the two clusters. **E** An alluvial diagram showcases the relationship between cluster affiliation, survival status, and clinical stage in LUAD patients

Utilizing unsupervised clustering on the 24 ICSMRGs, we aimed to uncover previously unidentified subtypes associated with iron, copper, and sulfur metabolism in LUAD. The selection of the optimal number of clusters (k = 2) revealed a notable divergence among groups, indicating a clear classification of LUAD patients into two distinct groups (Fig. 2A, B). Across four cohorts, the differential expression of these 24 ICSMRGs in two Clusters maintained homogeneity (Fig. 2C). Besides, we found that patients assigned to Cluster 1 exhibited significantly better prognoses compared to those in Cluster 2 (Fig. 2D), with individuals in Cluster 2 demonstrating more advanced clinical characteristics (Fig. 2E). Thus, our findings reveal two distinct molecular subtypes associated with iron, copper, and sulfur metabolism, potentially unveiling underlying biological heterogeneity in LUAD.

### The iron, copper and sulfur-metabolic index (ICSMI) was constructed

**Fig. 3:**
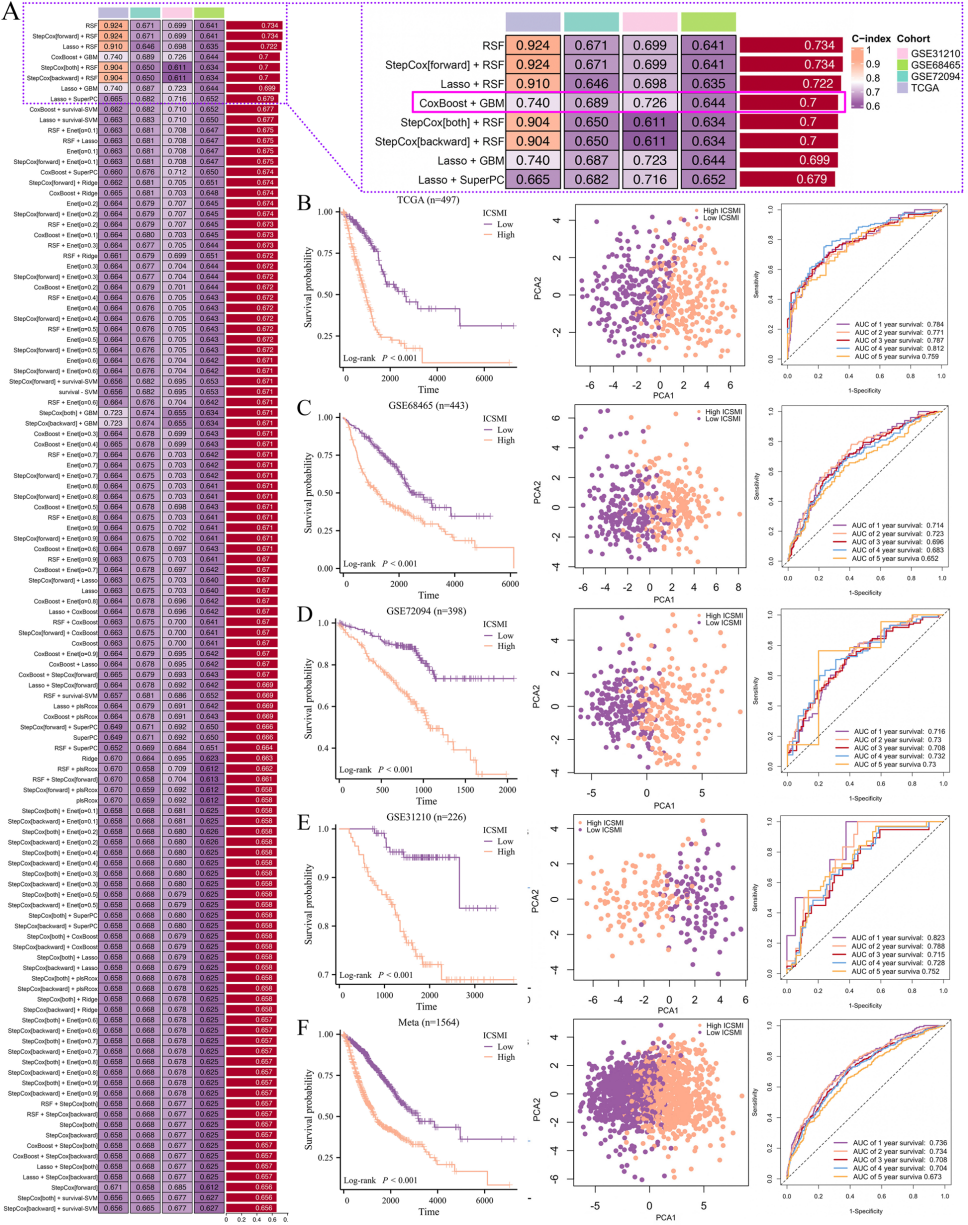
Integrated machine-learning for developing ICSMI. **A** A pragmatic evaluation of 114 distinct models was carried out through C-Index assessment across four independent cohorts. **B**-**F** Comparative analyses of prognostic variations, PCA, and time-ROC analysis were conducted between high- and low-ICSMI groups in TCGA (**B**), GSE68465 (**C**), GSE31210 (**D**), GSE72094 (**E**), and Meta (**F**) cohorts

The construction of the Iron, Copper, and Sulfur-Metabolic Index (ICSMI) was initiated by utilizing a machine learning-driven approach based on the 24 prognostic ICSMRGs. Using the TCGA dataset as training set, we developed 114 prediction models and assessed their performance on three independent validation sets (GSE68465, GSE72094, and GSE31210). While certain models, such as 'RSF,' 'Stepcox [forward] + RSF,' and 'Lasso + RSF,' exhibited high C-Index values in the TCGA dataset, their performance diminished in the validation sets, indicating overfitting. To ensure consistent predictive power across all datasets, we selected the 'CoxBoost + GBM' composition, which yielded a model with an average C-Index of 0.7 across all four datasets (C-Index: TCGA-0.740; GSE72094-0.689, GSE31210-0.726; GSE68465-0.644; Fig. 3A). The CoxBoost algorithm selected 10 ICSMRGs (Supplementary Fig. 1A), and the GBM algorithm evaluated their relative influence within the model (Supplementary Fig. 1B, Table S3), resulting in a GBM model comprising these 10 ICSMRGs (Fig. 3A). Kaplan–Meier analysis demonstrated a significant impact of all 10 ICSMRGs on the prognosis of LUAD patients (Supplementary Fig. 1E). Using the expression of these 10 ICSMRGs weighted by their relative influence, the model computed a riskscore for each individual, termed as ICSMI.

The median ICSMI was utilized to stratify patients into two distinct groups. Patients in the high-ICSMI group exhibited significantly poorer prognoses compared to those in the low-ICSMI group, not only within the TCGA training set (Fig. 3B) but also in three external validation cohorts, namely GSE68465 (Fig. 3C), GSE72094 (Fig. 3D), GSE31210 (Fig. 3E), and Meta (Fig. 3F). Additionally, PCA analysis revealed noticeable differences between individuals with high or low ICSMI across all datasets, and time-ROC curves illustrate the commendable predictive capabilities of ICSMI for predicting patients' prognosis, with high AUC values (Fig. 3B-F).

### A significant correlation is evident between ICSMI and clinical features of LUAD patients

**Fig. 4:**
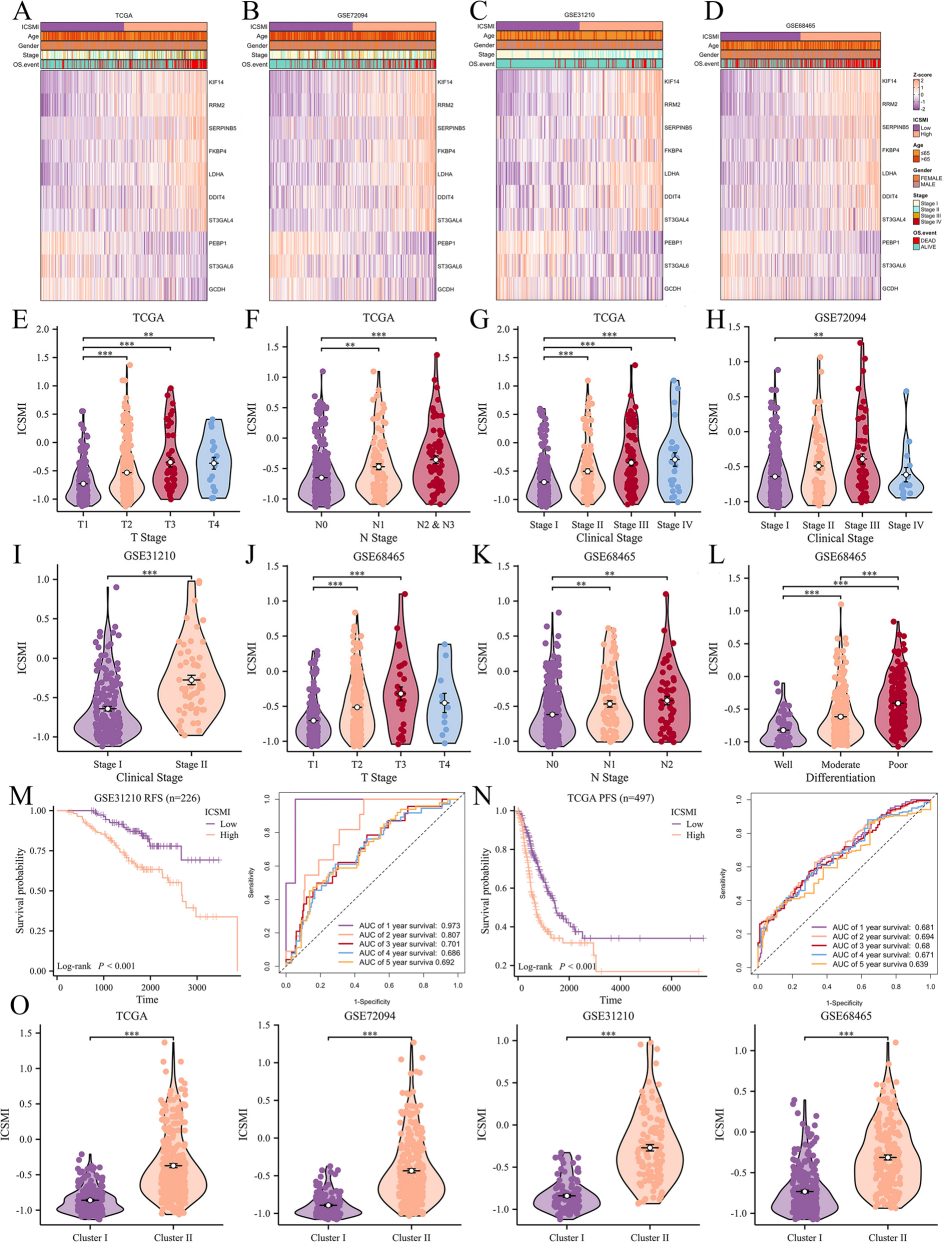
A robust correlation is evident between ICSMI and clinical attributes among LUAD patients. **A**-**D** Heatmaps illustrate expression profiles of 10 ICSMRGs across four datasets. **E**–**G** Individuals with high ICSMI in the TCGA Cohort exhibit increased prevalence of advanced T (**E**), N (**F**), and clinical stage (**G**). (H-I) ICSMI in LUAD patients escalates with clinical stage advancement in GSE72094 (**H**) and GSE31210 (I) cohorts. **J**-**L** In the GSE68465 cohort, patients' ICSMI elevates with T (**J**) and N (**K**) stage progression, alongside poorer differentiation (**L**). **M** In the GSE31210 cohort, patients' RFS declines with increasing ICSMI. **N** In the TCGA cohort, patients' PFS diminishes with rising ICSMI. **O** Significantly, all four cohorts demonstrate higher levels of ICSMI among patients assigned to Cluster 2

Heatmaps depict the transcriptional profiles of the 10 ICSMRGs comprising ICSMI across four distinct datasets: TCGA, GSE72094, GSE31210, and GSE68465 (Fig. 4A-D). In the TCGA cohort, ICSMI in LUAD patients increases with the progression of T (Fig. 4E), N (Fig. 4F), and clinical stage (Fig. 4G). Similarly, in the GSE72094 (Fig. 4H) and GSE31210 (Fig. 4I) cohorts, ICSMI increases with clinical stage progression. In the GSE68465 cohort, ICSMI elevates with advanced T (Fig. 4J) and N (Fig. 4K) stage, with a significant association observed with LUAD histology (Fig. 4L). Particularly, in poorly differentiated LUAD tissues, ICSMI is highest, followed by moderately differentiated tissues, and lowest in highly differentiated tissues. Furthermore, our investigation unveils a negative correlation between ICSMI and patients' Relapse-Free Survival (RFS) in the GSE31210 cohort (Fig. 4M), accompanied by a parallel decrease in patients' Progress-Free Survival (PFS) within the TCGA cohort (Fig. 4N). These findings underscore the potential utility of ICSMI as a prognostic biomarker in LUAD. Lastly, across all four cohorts, patients assigned to Cluster 2 exhibit markedly higher ICSMI values compared to those allocated to Cluster 1 (Fig. 4O), indicating a notable association between Iron, Copper, and Sulfur-Metabolism (ICSM) related molecular subtypes and ICSMI.

### Comparison the predictive efficacy of ICSMI with existing characteristics

**Fig. 5:**
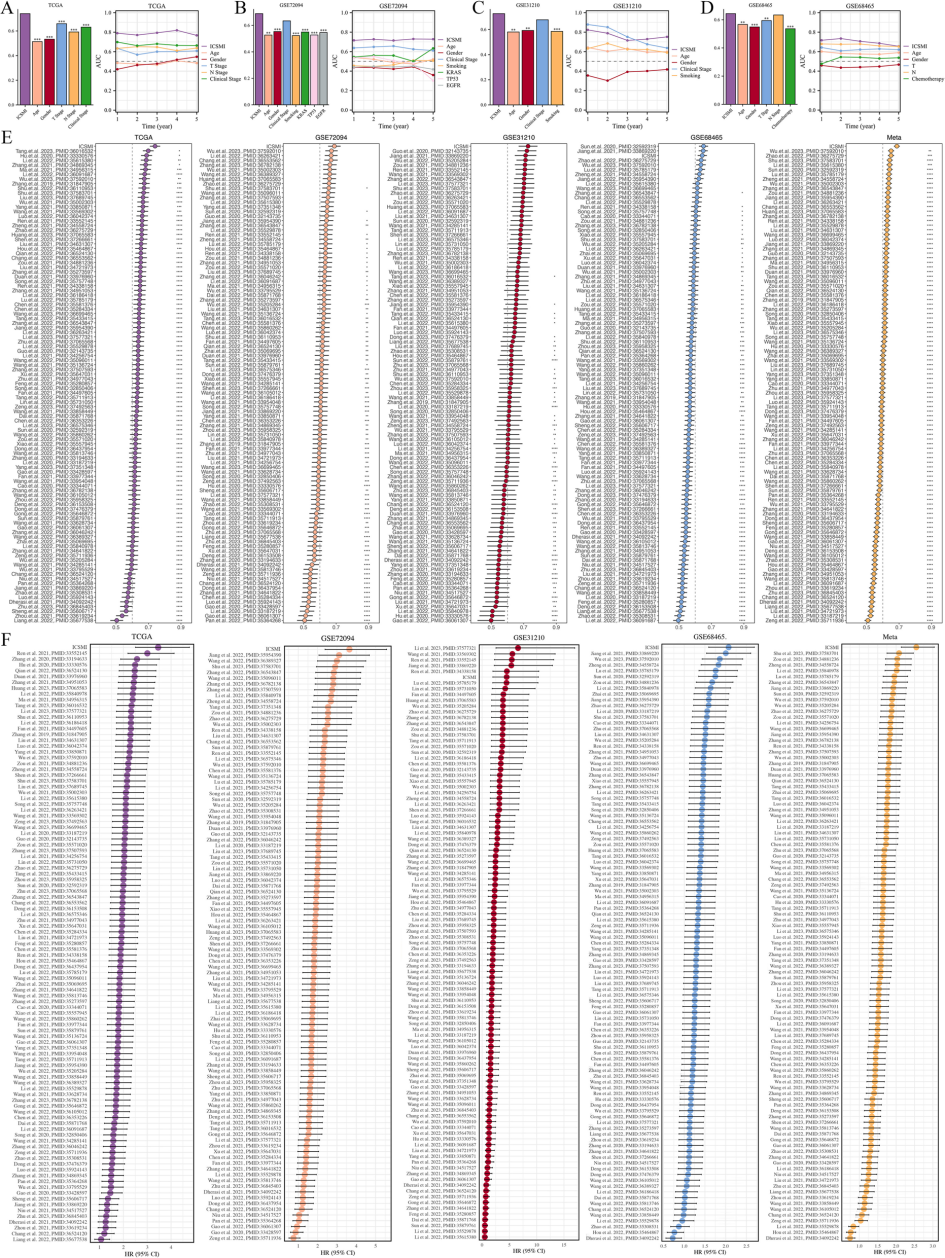
Assessing the predictive capability of ICSMI. **A**-**D** Contrasting the C-Index and AUC value of ICSMI with clinical factors in the TCGA (**A**), GSE72094 (**B**), GSE31210 (**C**), and GSE68465 (**D**) cohorts. **E** Comparing the C-Index of ICSMI with 102 previously published signatures. **F** Comparing the HR-Value of ICSMI with 102 previously published signatures

To assess the predictive efficacy of ICSMI compared to traditional clinical variables in LUAD patients, we conducted an analysis of C-index and AUC values for each factor (Fig. 5A-D). Notably, ICSMI exhibited superior predictive performance compared to most clinical markers, indicating its enhanced efficiency. Additionally, we evaluated the prognostic potential of ICSMI against established LUAD models by integrating data from 102 prior studies incorporating various biologically relevant features like apoptosis, EMT, ferroptosis, cuproptosis, necroptosis, and ICD (Table S6). Impressively, ICSMI consistently displayed the highest C-index (Fig. 5E) and HR value (Fig. 5F) across multiple cohorts, surpassing the majority of existing models. These findings collectively highlight ICSMI as a more effective prognostic model for LUAD.

### Nomogram’s development and validation

**Fig. 6:**
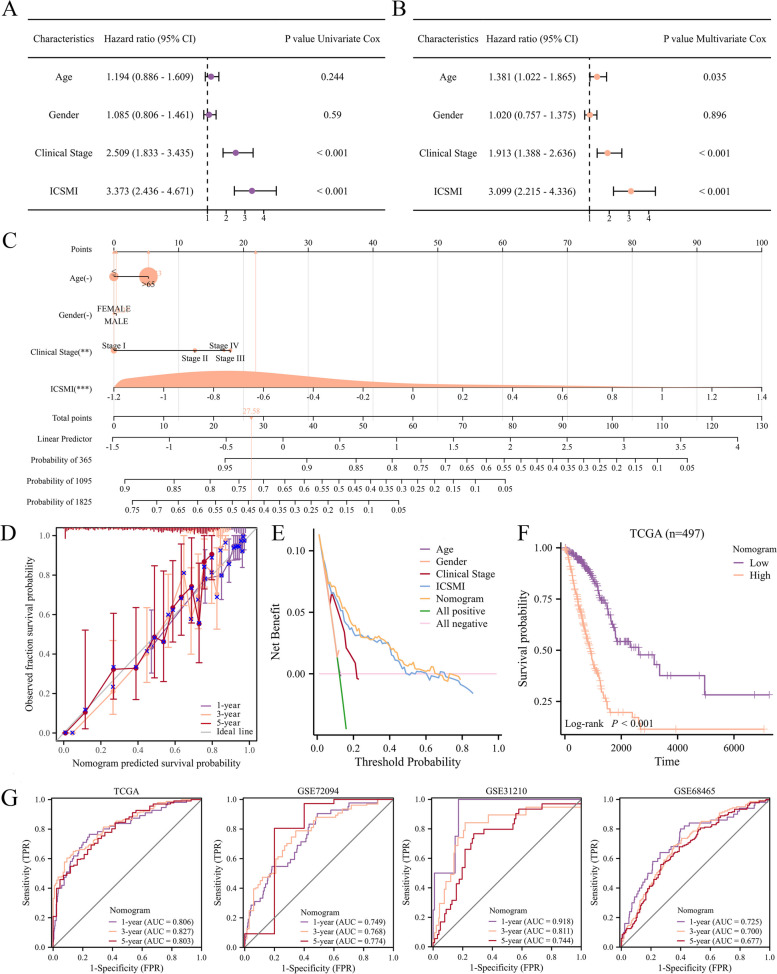
Developing a nomogram. **A**-**B** Uni- (**A**) and multi- (**B**) vadiate cox regression affirm ICSMI as an independent prognostic determinant. **C**, **D** Creation of the nomogram (**C**) and its calibration curve (**D**) showcase its predictive accuracy. **E** Decision curve analysis (DCA) curves indicate the superior prognostic performance of the nomogram for LUAD patients. **F** Patients with elevated nomogram scores exhibit poorer prognoses. **G** ROC curves across four cohorts underscore the remarkable predictive prowess of the nomogram

To validate the independent predictive value of ICSMI, we conducted univariate and multivariate Cox regression analyses. After excluding the influence of clinical variables, our analysis unequivocally established ICSMI as a significant predictor of LUAD patient prognosis, confirming its status as an independent prognosticator not only within the TCGA cohort (Fig. 5A, B), but also within the GSE68465, GSE72094, and GSE31210 cohorts (Tables 1, 2 and 3). Integrating ICSMI with clinical markers such as age, gender, and clinical stage, we developed a nomogram for forecasting LUAD patient prognosis (Fig. 6C). Our model achieved a C-index value of 0.768, with calibration plots confirming its accuracy in estimating 1-, 3-, and 5-year survival probabilities (Fig. 6D). Additionally, employing decision curve analysis (DCA), our nomogram model demonstrated superiority over alternative predictors (Fig. 6E). Notably, significant survival differences were observed between high- and low-nomogram score groups (Fig. 6F). Furthermore, AUC values across four cohorts revealed the remarkable precision of our nomogram in predicting 1-, 3-, and 5-year survival prospects for LUAD patients (Fig. 6G).

**Table 1:** Uni- and Multi- variate Cox analysis performed in GSE68465 cohort

Factors	Univariate analysis		Multivariate analysis	
	**HR value (95% CI)**	***P***	**HR value (95% CI)**	***P***
**ICSMI**	2.018 (1.544—2.637)	** < 0.001**	1.758 (1.342—2.304)	** < 0.001**
**Age**	0.653 (0.501—0.851)	**0.002**	0.608 (0.465—0.795)	** < 0.001**
**Gender**	0.688 (0.528—0.897)	**0.006**	0.754 (0.572—0.992)	**0.044**
**T Stage**	2.770 (1.890—4.058)	** < 0.001**	2.208 (1.494—3.261)	** < 0.001**
**N Stage**	2.814 (2.155—3.675)	** < 0.001**	2.678 (2.031—3.533)	** < 0.001**
**Chemotherapy**	1.677 (1.254—2.244)	** < 0.001**	1.428 (1.048—1.946)	**0.024**

**Table 2:** Uni- and Multi- variate Cox analysis performed in GSE72094 cohort

Factors	Univariate analysis		Multivariate analysis	
	**HR value (95% CI)**	***P***	**HR value (95% CI)**	***P***
**ICSMI**	3.385 (2.246—5.103)	** < 0.001**	2.861 (1.860—4.399)	** < 0.001**
**Age**	0.702 (0.458—1.076)	0.105	0.729 (0.472—1.126)	0.154
**Gender**	1.552 (1.072—2.246)	**0.020**	1.542 (1.037—2.292)	**0.033**
**Clinical Stage**	2.562 (1.720—3.816)	** < 0.001**	2.332 (1.540—3.532)	** < 0.001**
**Smoking**	1.377 (0.601—3.157)	0.450	0.911 (0.384—2.164)	0.833
**KRAS**	1.456 (1.001—2.118)	**0.049**	1.026 (0.691—1.521)	0.900
**TP53**	1.235 (0.820—1.860)	0.313	0.892 (0.584—1.364)	0.599
**EGFR**	0.262 (0.096—0.710)	**0.008**	0.451 (0.159—1.280)	0.135

**Table 3:** Uni- and Multi- variate Cox analysis performed in GSE31210 cohort

Factors	Univariate analysis		Multivariate analysis	
	**HR value (95% CI)**	***P***	**HR value (95% CI)**	***P***
**ICSMI**	4.503 (1.965—10.319)	** < 0.001**	3.330 (1.382—8.026)	**0.007**
**Age**	1.025 (0.977—1.075)	0.306	1.042 (0.993—1.092)	0.091
**Gender**	0.658 (0.338—1.281)	0.219	0.922 (0.360—2.362)	0.866
**Clinical Stage**	4.232 (2.175—8.236)	** < 0.001**	2.850 (1.409—5.765)	**0.004**
**Smoking**	0.611 (0.312—1.195)	0.150	0.834 (0.321—2.168)	0.710

### ICSMI has significantly relationships with TME

**Fig. 7:**
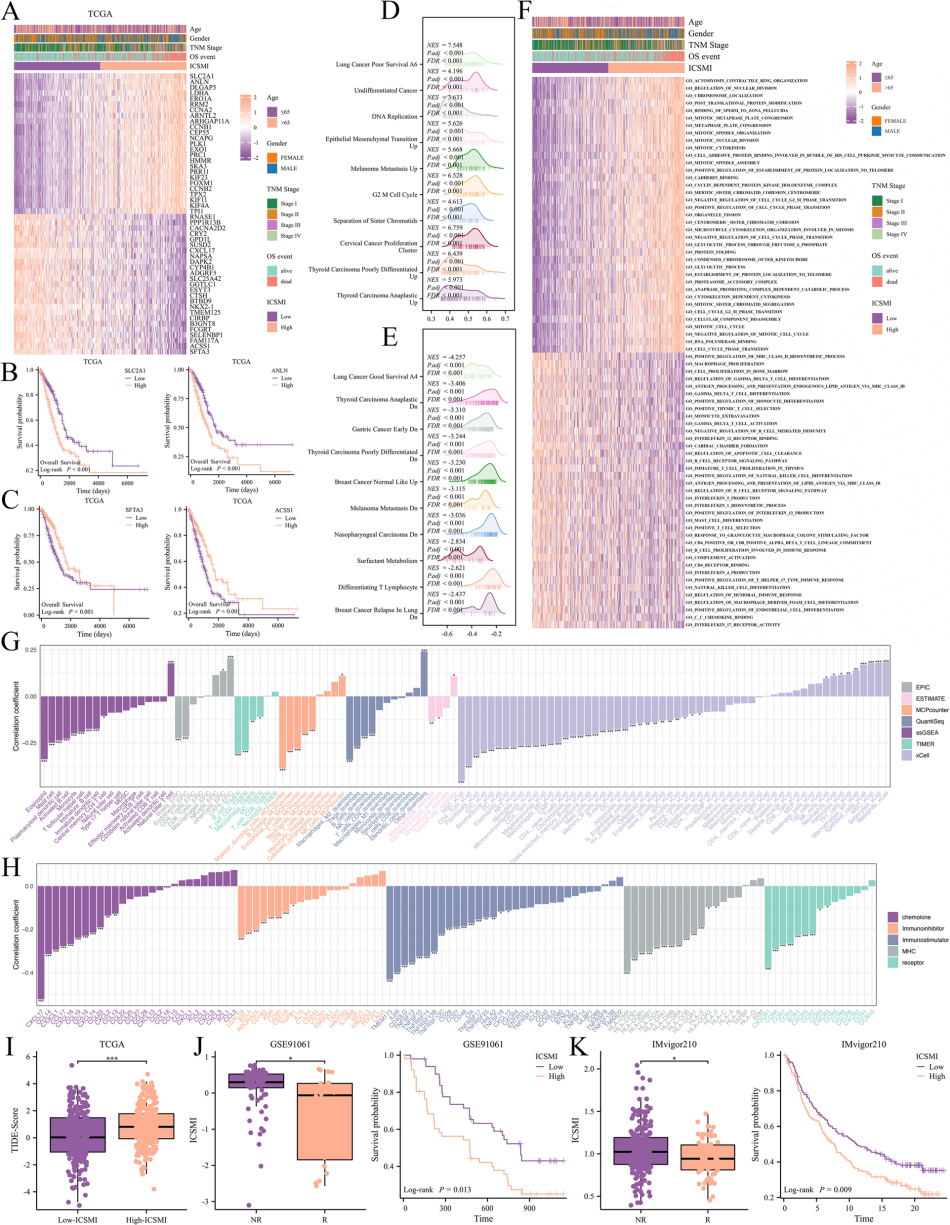
Uncover the potential involvement of ICSMI in the TME. **A** To identify the most highly correlated genes with ICSMI, a heatmap of the top 50 genes was generated. **B**, **C** Elevated expression of SLC2A1 and ANLN adversely affected the prognosis for LUAD patients, while elevated expression of SFTA3 and ACSS1 improved the prognosis for LUAD patients. **D**, **E** GSEA analysis unveiled the functional enrichment of genes positively (**D**) or negatively (**E**) correlated with ICSMI. **F** GSVA analysis disclosed the gene sets with heightened activity in high- and low- ICSMI groups. **G** An inverse correlation was observed between ICSMI and the infiltration of most immune cells. **H** ICSMI was found to be negatively correlated with the expression of TME modulators. **I** Patients in the low-ICSMI group exhibited lower TIDE scores. **J**, **K** Responders to immunotherapy were found to have lower ICSMI levels, and patients receiving immunotherapy with lower ICSMI tended to have better overall survival outcomes in the GSE91061 (**J**) and IMvigor210 (**K**) cohorts

Next, we performed investigations into the underlying mechanism behind the remarkable predictive capability of ICSMI, particularly its relationships with the Tumor Microenvironment (TME). In the TCGA cohort, differential analysis highlighted genes with differing expression levels between groups with high and low ICSMI levels (Table S4). The top 50 genes, showing the most significant expression differences, were visually depicted (Fig. 7A). Additionally, we delved into the impact of the two most up-regulated genes in the high-ICSMI group (SLC2A1, ANLN) and the two most up-regulated genes in the low-ICSMI group (SFTA3, ACSS1) on LUAD patient prognosis. Elevated expression of ANLN and SLC2A1 was associated with poorer prognosis, while increased expression of SFTA3 and ACSS1 indicated better prognosis (Fig. 7B, C). This suggests that ICSMI serves as a risk factor for LUAD, with high expression correlating with adverse outcomes. GSEA analysis unveiled that genes positively correlated with ICSMI were predominantly involved in malignant features, while genes negatively correlated with ICSMI were associated with benign features (Fig. 7D, E). GSVA analysis further supported these findings, with gene sets linked to malignant features showing higher activity in the high-ICSMI group, while those related to benign phenotypes exhibited greater activity in the low-ICSMI group (Fig. 7F).

Analysis across seven algorithms revealed a negative correlation between ICSMI and the majority of immune cells, while it correlated positively with epithelial cells and CAFs (Fig. 7G). Moreover, ICSMI displayed a negative correlation with several immune checkpoint molecules (Fig. 7H). Notably, individuals in the low-ICSMI group exhibited more pronounced 'immuno-hot' features, suggesting potential responsiveness to immunotherapeutic interventions. This was supported by lower TIDE scores in the low-ICSMI group (Fig. 7I), indicating improved response to immunotherapy. Furthermore, analysis of GSE91061 and IMvigor210 datasets showed that responders to ICB therapy had lower ICSMI values compared to non-responders, and patients in the low-ICSMI group undergoing immunotherapy demonstrated significantly better clinical outcomes (Fig. 7J).

### Exploring ICSMI at single-cell level

**Fig. 8:**
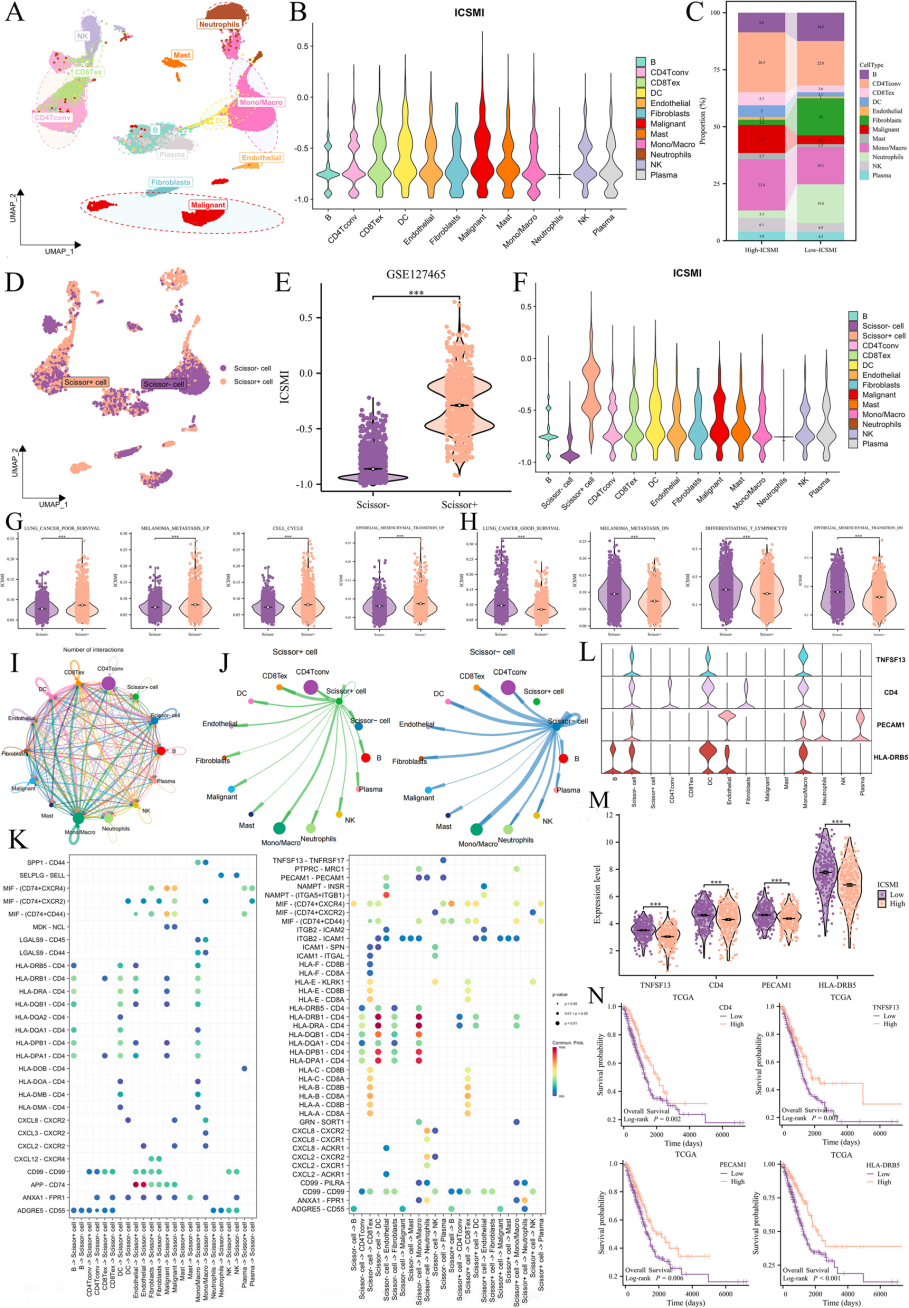
Utilizing the Scissor algorithm to segregate high- and low- ICSMI characteristics within the single-cell dataset. **A** Mapping the distribution of 12 cell populations within the tumor microenvironment (TME) using the UMAP plot. **B** Violin plot illustrating the spectrum of ICSMI levels across diverse cell types. **C** The high-ICSMI group showed an elevated proportion of malignant cells. **D** Identified 1566 high-ICSMI cells (Scissor +) and 2151 low-ICSMI cells (Scissor-). **E** Notably, Scissor + cells exhibited substantially higher ICSMI levels compared to Scissor-. **F** Among all cell types, Scissor ± displayed the most varied ICSMI levels. **G**, **H** Scissor + demonstrated heightened scores indicative of a malignant phenotype (**G**), while Scissor- scored higher for a benign phenotype (**H**). **I** Comprehensive depiction of cellular communication networks. **J** Scissor- showed robust signaling transmission capabilities within the TME. **K** Comparison of Scissor ± cells' proficiency in signal reception and transmission within the TME. **L** Scissor- exhibited specific molecule expression tailored to pair with ligands from other cells. **M** These molecules exhibited elevated expression levels in low-ICSMI patients. **N** The four molecules specifically expressed by Scissor- are identified as protective factors for LUAD

In the Bulk-dataset, ICSMI was an independent risk factor for LUAD patients. We were also very interested in the effects of ICSMI at the cellular level, so we analyzed the single-cell RNA-sequencing dataset. In the GSE127465 dataset, we identified 12 distinct cell populations (Fig. 8A) and computed the ICSMI for each cell. Remarkably, malignant cells exhibited the highest ICSMI values (Fig. 8B), with the high-ICSMI group showing a higher proportion of malignant cells (Fig. 8C). To pinpoint the cellular sources underlying the clinical manifestation associated with high-ICSMI, we utilized the "scissor" package to correlate bulk RNA-sequencing data with single-cell RNA-sequencing data. This algorithm autonomously selected cells exhibiting extraordinary concordance with the targeted phenotype. We designated high-ICSMI and low-ICSMI patient states as primary phenotypes, facilitating the identification of a comprehensive collection of 1566 high-ICSMI cells (Scissor +) and 2151 low-ICSMI cells (Scissor-, Fig. 8D). Notably, Scissor + cells exhibited significantly higher ICSMI values compared to Scissor- cells (Fig. 8E), with Scissor + cells displaying the highest ICSMI among all cell types, while Scissor- cells had the lowest (Fig. 8F). These findings indicate our success in identifying cells in the single-cell dataset that represent different ICSMI states.The AUCell algorithm[35] was used to calculate enrichment scores for multiple gene sets, and we compare them between Scissor + and Scissor-. Scissor + scored significantly higher than Scissor- for four malignant phenotype including 'Lung Cancer Poor Survival’, ‘Melanoma Metastasis UP’, ‘Cell Cycle’, and 'Epithelial Mesenchymal Transition UP' (Fig. 8G); while Scissor- scored significantly higher that Scissor + for four benign phenotypes including ‘Lung Cancer Good Survival’, ‘Melanoma Metastasis DN’, ‘Differentiating T Lymphocyte’, and 'Epithelial Mesenchymal Transition DN' (Fig. 8H). This result is consistent with the GSEA analysis performed in the Bulk-data set.

Following this, we delved into the intercellular communication dynamics. The communication network among all cells is depicted in Fig. 8I. Interestingly, compared to Scissor + cells, Scissor- cells exhibited higher effectiveness in transmitting signals to other cells (Fig. 8J). Furthermore, when comparing the ability of Scissor + and Scissor- to both receive and transmit signals, Scissor- demonstrated greater activity in communicating with other cells within the TME (Fig. 8 K). We observed that Scissor- specifically expressed various receptors/ligands to interact with ligands/receptors from other cells, a capability not shared by Scissor + . Notably, Scissor- expressed TNFSF13, HLA-DRB5, CD4, and PECAM1 specifically (Fig. 8L) to exchange signals with cells such as CD4Tconv, DC, Monocytes, and Fibroblasts. Analysis of bulk TCGA data revealed that the expression of these four molecules, specifically expressed by Scissor-, was significantly higher in the low-ICSMI group (Fig. 8M), and all of them are protective factors for LUAD (Fig. 8N).

In summary, the characteristics of Scissor ± in the single-cell dataset align with those of ICSMI-high/low in the bulk dataset, thus corroborating our conclusions from different perspectives.

### Comparing the different SNV and CNV event between two ICSMI groups

**Fig. 9:**
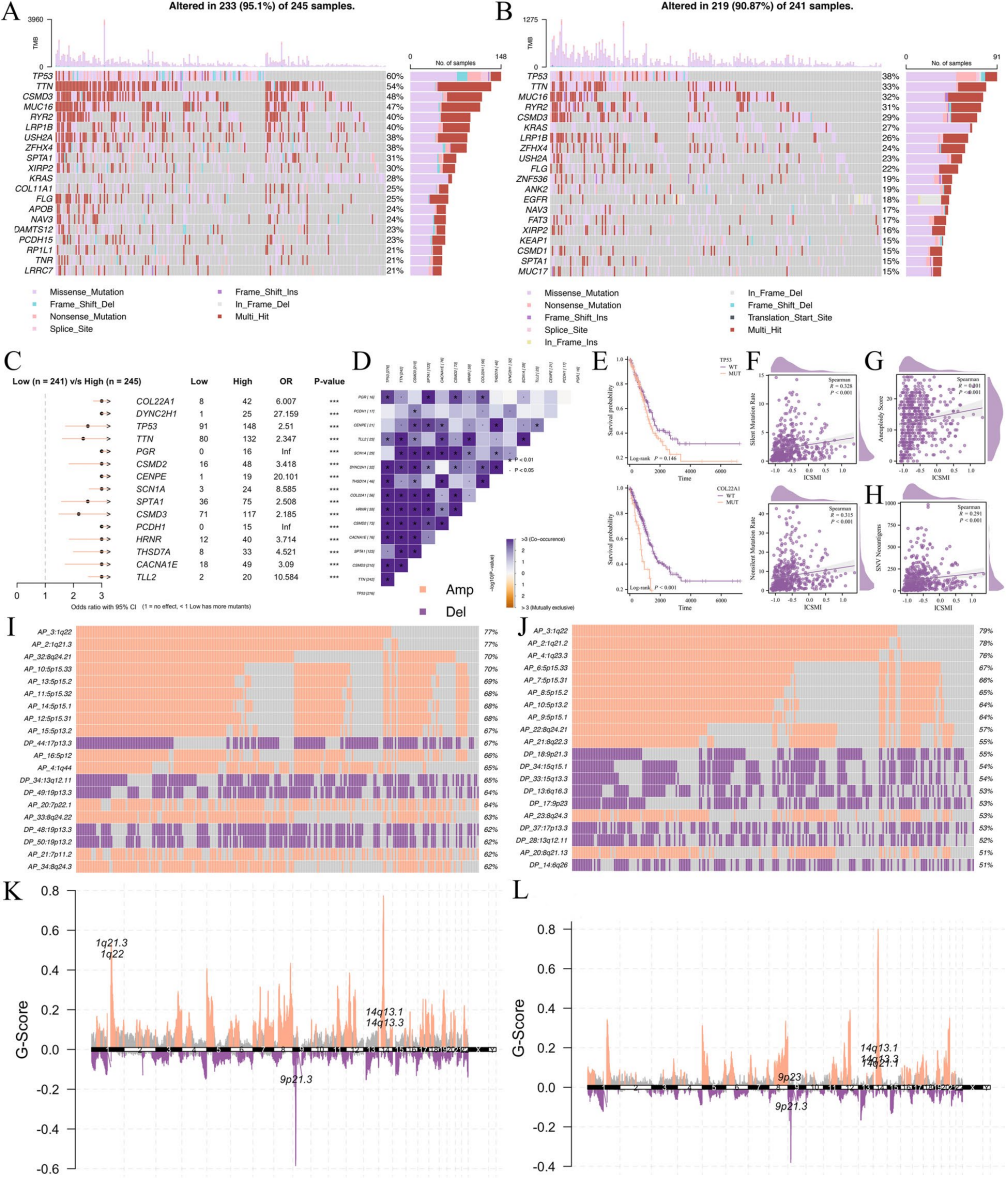
The genetic landscape displayed significant differences between the two ICSMI groups. **A**, **B** Comparison of somatic mutation frequencies in high- (**A**) and low- (**B**) ICSMI patient cohorts. (**C**, **D**) Identification of the top 15 differentially mutated genes between the two groups (**C**), accompanied by significant co-occurrences among them (**D**). **E** Mutations in COL22A1 and TP53 are associated with poorer prognosis. **F**, **H** Positive correlation of ICSMI with two forms of gene mutation (**F**), aneuploidy score (**G**), and SNV neoantigen (H). (I-J) Analysis of the top 20 copy number variations (CNVs) in the high- (**I**) and low- (**J**) ICSMI groups. **K**, **L** Visualization of patients' G-scores using chromosomal plots in the high- (**K**) and low- (**L**) ICSMI groups

We also conducted multi-omics analyses to compare the genetic landscape between the high-ICSMI and low-ICSMI groups. Initially focusing on the top 20 genes with the highest mutation frequency, we visually depicted the disparities between these groups (Fig. 9A, B). The followed examination showed that the top 15 genes with the most notable differences in mutation frequency between the high- and low-ICSMI groups exhibited higher frequencies within the former (Fig. 9C). Additionally, we identified co-mutation relationships among these genes (Fig. 9D). A detailed investigation was conducted on the most significant mutation differences between the two groups, particularly in the genes COL22A1 and TP53. We investigated the prognostic implications of mutations in these genes and discovered that such mutations were linked to adverse outcomes for patients with LUAD (Fig. 9E). Moreover, ICSMI exhibited a strong positive correlation with various forms of gene mutations (Fig. 9F), and significantly correlated with aneuploidy score (Fig. 9G) and SNV neoantigen (Fig. 9H).

Furthermore, our analysis revealed considerable divergence in CNV events between the two ICSMI groups (Fig. 9I, J). Patients in the high-ICSMI group displayed a higher frequency and more complex array of CNV events, whereas those in the low-ICSMI group exhibited fewer and less elaborate CNV events. ChromPlots further demonstrated that patients in the high ICSMI group had higher G-scores compared to those in the low ICSMI group (Fig. 9 K, L), suggesting a propensity for malignant features among high-ICSMI patients with LUAD.

### Exploration of latent agents targeting ICSMI

**Fig. 10:**
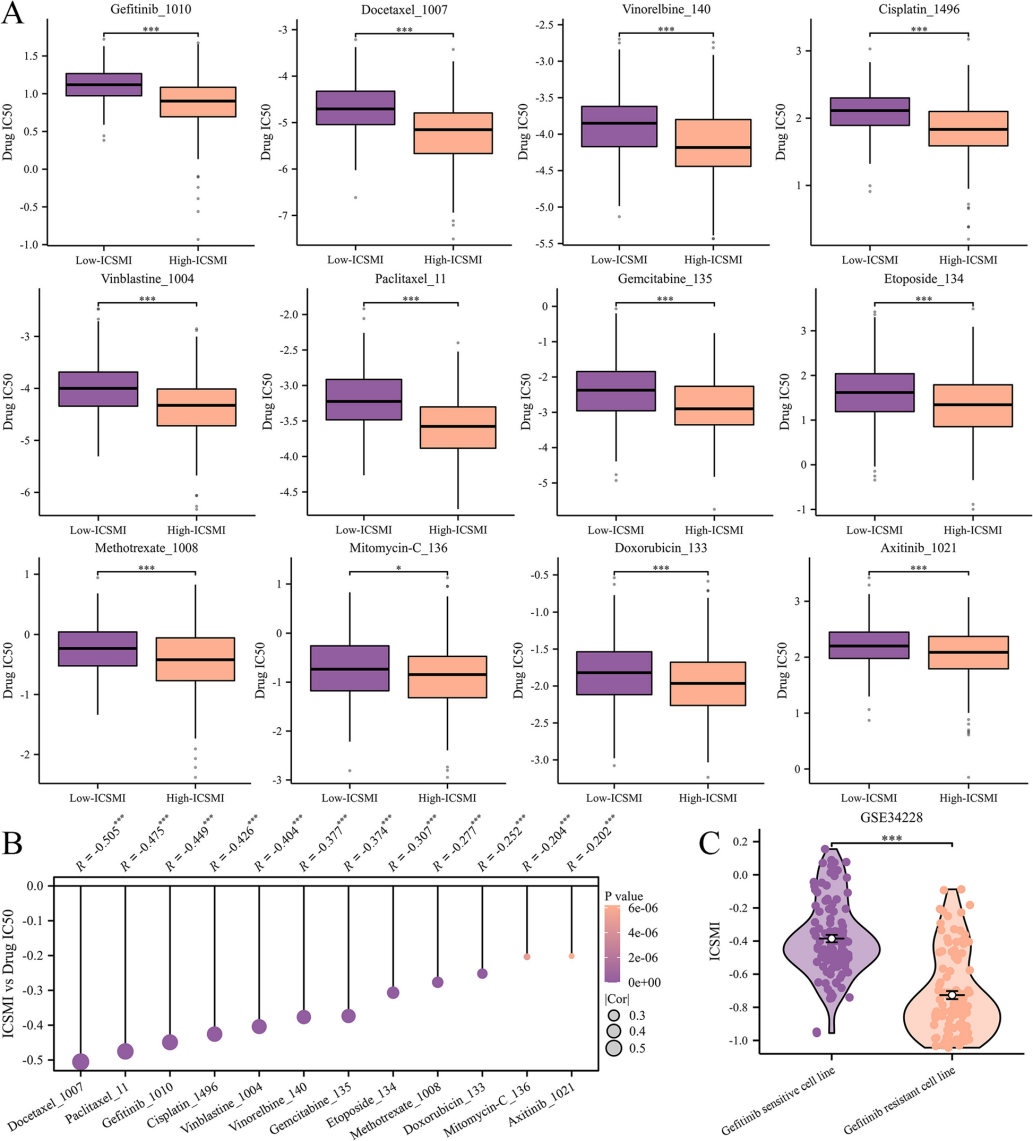
Chemotherapy and targeted therapy might exhibit heightened efficacy in high-ICSMI patients. **A** Variations in IC50 values of 12 frequently prescribed drugs between high- and low- ICSMI cohorts are evident. **B** Correlation between ICSMI levels and drugs' IC50 values is observed. **C** Differences in ICSMI are apparent between cell lines sensitive versus resistant to gefitinib

To uncover potential therapeutic avenues against ICSMI, we examined the connection between ICSMI and commonly used drugs for treating LUAD. Among the twelve medications analyzed, their IC50 values were notably lower in the high-ICSMI cohort compared to the low-ICSMI subset (Fig. 10A). Additionally, correlation analysis indicated a negative correlation between ICSMI and the IC50 values of these drugs (Fig. 10B), suggesting that these medications may be more effective in patients with higher ICSMI levels. Particularly noteworthy was the observation that LUAD cell lines sensitive to gefitinib exhibited significantly higher ICSMI levels compared to gefitinib-resistant cell lines (Fig. 10C), providing further support for our findings.

### Exploring the prognostic value of ICSMI in other cancers besides LUAD

**Fig. 11:**
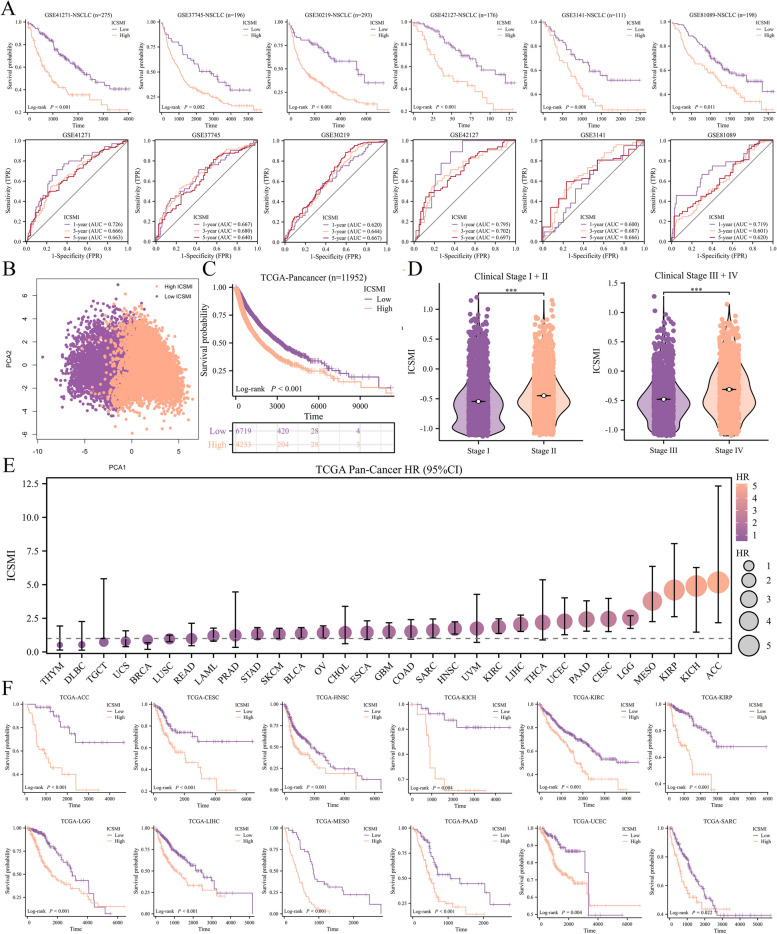
ICSMI’s value in pan-cancer cohort. **A** 6 independent cohorts affirmed that NSCLC patients with higher ICSMI had poorer prognosis. **B** The PCA plot uncovering distinct characteristics of different ICSMI group patients. **C** Patients with higher ICSMI had poorer OS. **D** ICSMI increased with Stage progressed. **E** ICSMI is a risk factor for most cancer patients. **F** High ICSMI leads to poorer prognosis in 12 types of cancer patients

Given the impressive performance of ICSMI in predicting the prognosis of LUAD patients, we are highly interested in exploring its value in predicting the prognosis of other types of cancer. First, we investigated the prognostic value of ICSMI in patients with other types of NSCLC. We selected six GEO datasets containing information on various types of NSCLC patients and calculated ICSMI for each patient. The results indicate that, across the six independent datasets, patients with high ICSMI have a worse prognosis compared to those with low ICSMI, and ROC curves showed that ICSMI also had good predictive power (Fig. 11A). Next, we obtained the TCGA-Pancancer dataset, which contains information about over 11,000 patients with 33 different types of cancer. PCA analysis shows that patients between high and low ICSMI groups exhibit distinct features (Fig. 11B). Notably, patients in the high ICSMI group have significantly lower OS than those in the low ICSMI group (Fig. 11C). Furthermore, as the clinical stage advances, ICSMI displays a gradually increasing trend (Fig. 11D). We then analyzed the prognostic value of ICSMI in each cancer individually. The HR value of ICSMI was found to be greater than 1 in most cancers, indicating that ICSMI is a risk factor for most cancer patients (Fig. 11E), specifically for those patients with ACC, CESC, HNSC, KICH, KIRC, KIRP, LGG, LIHC, MESO, PAAD, UCEC, SARC (Fig. 11F).

### Validation of hub ICSMRGs' expression by qRT-PCR

**Fig. 12:**
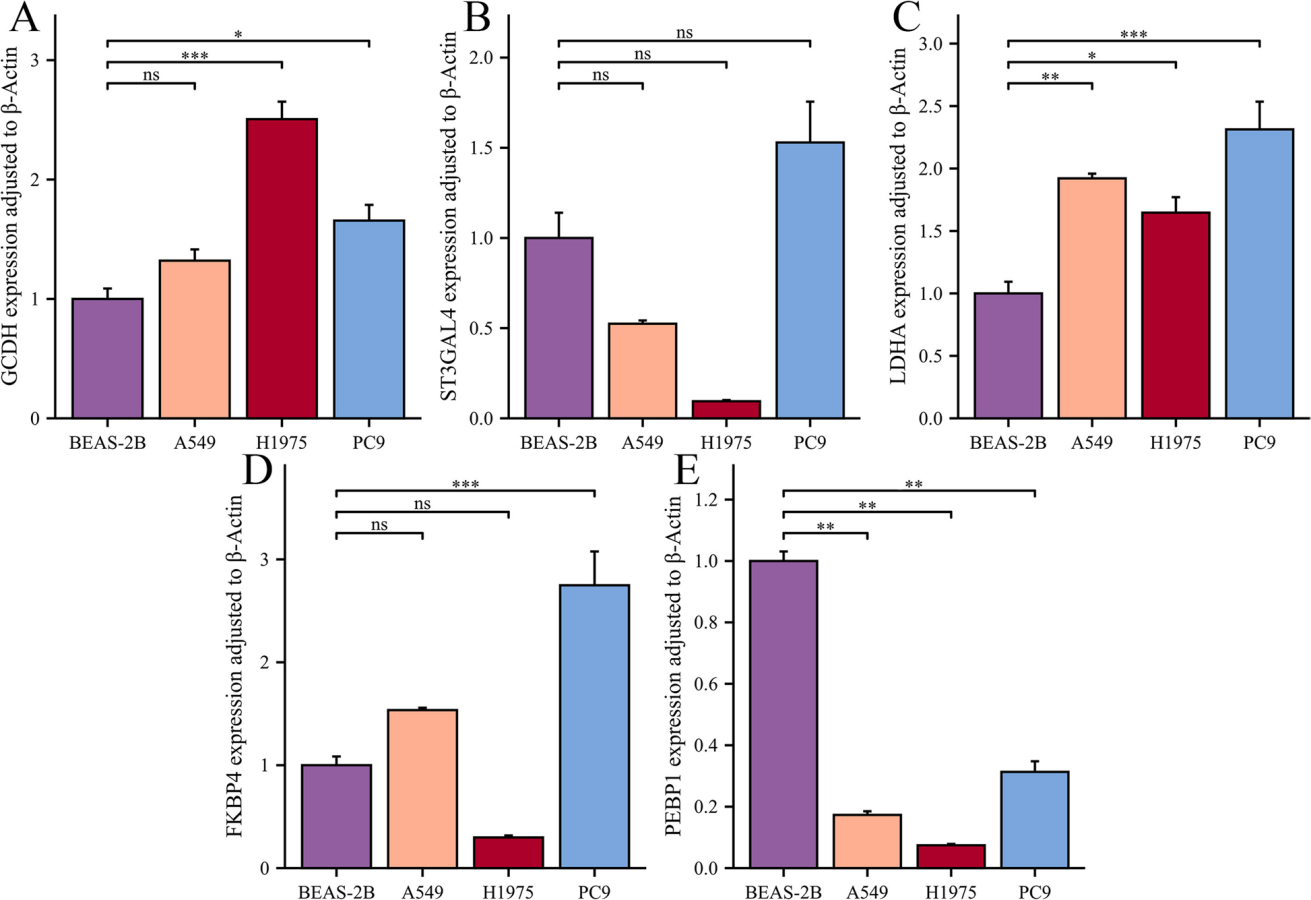
Validation of five hub ICSMRGs’ expression at cell lines. **A**-**E** The expression of GCDH (**A**), ST3GAL4 (**B**), LDHA (**C**), FKBP4 (**D**), and PEBP1 (**E**) in normal lung epithelial cell lines and three LUAD cell lines

To improve the credibility of our study, we opted to verify the expression of hub ICSMRGs. Our analysis revealed that within the GBM model comprising 10 ICSMRGs, five exhibited a relative influence exceeding 10 in the formation of ICSMI. Therefore, we defining these 5 ICSMRGs, namely GCDH, ST3GAL4, LDHA, FKBP4, and PEBP1 as hub ICSMRGs. In comparison with BEAS-2B, the expression of GCDH, LDHA, and FKBP4 shows an increasing trend in LUAD cell lines, while the expression of PEBP1 exhibits a decreasing trend. However, the expression of ST3GAL4 does not display significant differences between normal lung epithelial cells and LUAD cells. In summary, the expression trends of these five key ICSMRGs are generally consistent with the results analyzed in the TCGA dataset, laying the foundation for future functional experiments targeting these genes (Fig. 12).

### Knockdown of GCDH promoted LUAD cells’ migration and invasion

**Fig. 13:**
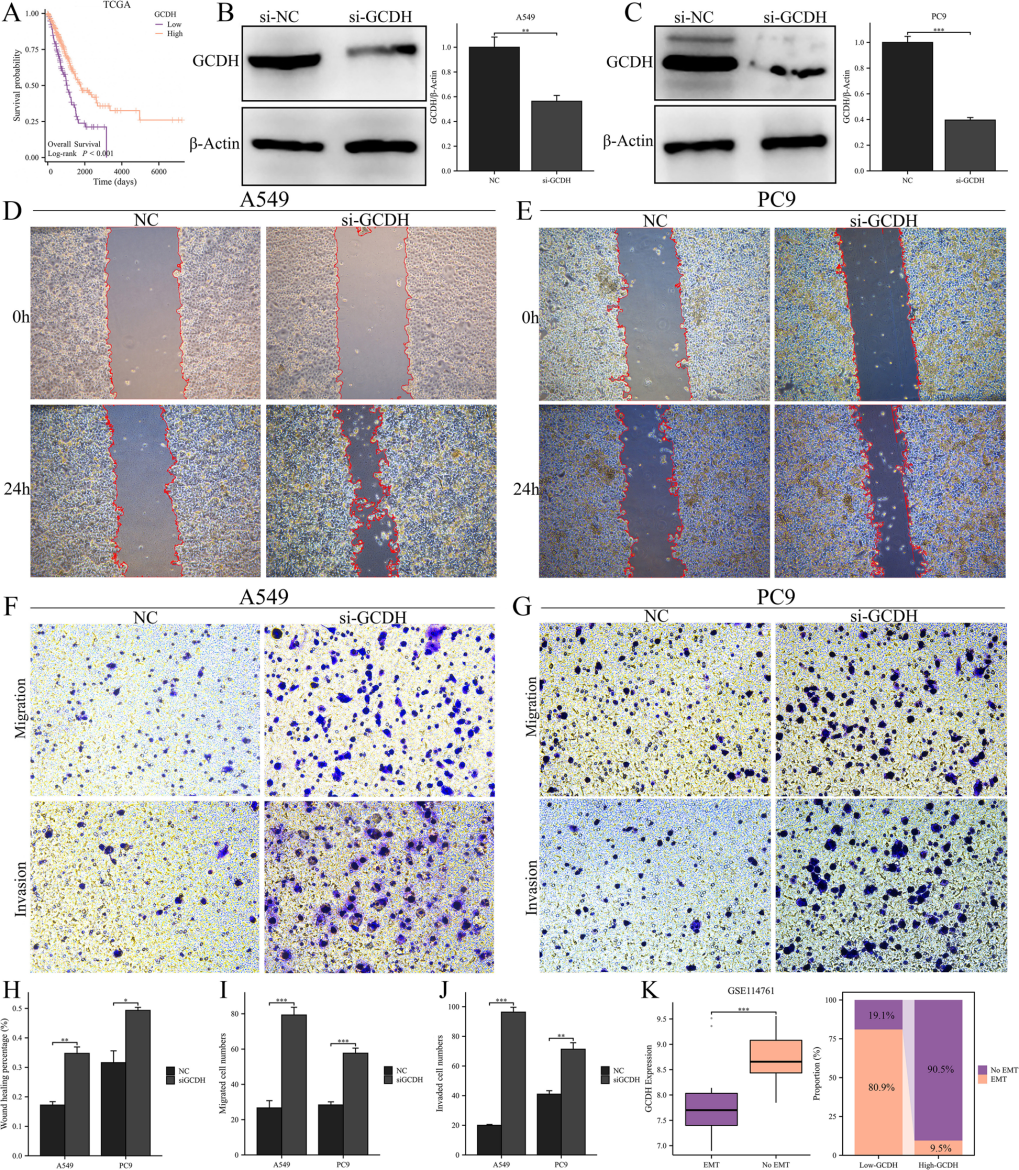
Knockdown of GCDH promotes LUAD cells' malignant phenotype. **A** High expression of GCDH confers better prognosis in the TCGA cohort. **B**, **C** The expression of GCDH was significantly reduced by siRNA’s knockdown in both A549 (**B**) and PC9 (**C**) cells. **D**, **E** Wound healing experiment performed in A549 (**D**) and PC9 (**E**) cells. **F**, **G** Transwell experiment performed in A549 (**F**) and PC9 (**G**) cells. **H** The result of the wound healing experiment was statistically significant. **I**, **J** Transwell experiment showed that knockdown of GCDH promotes LUAD cells’ migration (**I**) and invasion (**J**) ability. **K** Lung adenocarcinoma cells undergoing EMT had lower GCDH expression

Among the 10 ICSMRGs utilized in constructing ICSMI, GCDH exerts the most significant influence (Supplementary Fig. 1B). Furthermore, it's noteworthy that no study has yet explored the impact of GCDH on LUAD. Therefore, we decided to further explore the role of GCDH in LUAD. In our bioinformatics analysis, GCDH is a protective factor, which indicates that patients with higher expression of GCDH have a better prognosis (Fig. 13A). Next, we performed in vitro experiments to explore the potential phenotypes associated with GCDH. The expression of GCDH was significantly reduced by siRNA’s knockdown in both A549 (Fig. 13B) and PC9 (Fig. 13C) cells. Wound healing assay showed that knockdown of GCDH improved the migration abilities of both A549 (Fig. 13D) and PC9 (Fig. 13E) cells. In addition, the transwell assay also demonstrated that knocking out GCDH can promote the migration and invasion of A549 (Fig. 13F) and PC9 (Fig. 13G) cells. The results of the wound healing experiment (Fig. 13H) and the transwell experiment (Fig. 13I, J) both demonstrate statistical significance, indicating that knocking down GCDH significantly promotes the migration and invasion ability of A549 and PC9 cells. Epithelial-Mesenchymal Transition (EMT) is also a malignant phenotype closely associated with migration and invasion. Tumor cells undergoing EMT exhibit higher malignancy and are more prone to metastasis. Therefore, we investigated the relationship between GCDH and EMT. We downloaded the GSE114761 dataset from the GEO database, which includes EMT data from lung adenocarcinoma cell lines. We found that the expression of GCDH was significantly lower in cells undergoing EMT compared to those not undergoing EMT, and the proportion of cells undergoing EMT in the low-GCDH group is significantly higher than that in the high-GCDH group (Fig. 13K). Thus, low expression of GCDH may also promote EMT in LUAD cells.

Therefore, knocking out GCDH can promote the malignant phenotype of LUAD cells, and LUAD cells undergoing EMT exhibit lower GCDH expression, suggesting a protective role of GCDH in LUAD. This finding aligns with our bioinformatics analysis, indicating that patients with high GCDH expression have better prognoses. It further validates the conclusion of our study and the reliability of ICSMI.

## Discussion

The labyrinthine biology and varied clinical manifestations of lung cancer present significant hurdles for medical professionals. Yet, recent progress in high-throughput sequencing have paved the way for the discovery of new prognostic markers. These advancements empower healthcare providers to predict patient outcomes more precisely and tailor therapeutic approaches accordingly. Disruptions in the regulation of iron, copper, and sulfur metabolism can predispose individuals to various diseases. Precedent research has elucidated the function of genes implicated in iron and copper metabolism on LUAD TME, unequivocally establishing their impact upon treatment efficacy [22, 24, 36]. Conversely, the investigation of sulfur metabolism's involvement in LUAD pathogenesis remains relatively unexplored, and iron, copper, and sulfur-metabolism genes have not been combined together to create prognostic signatures. With an eye toward unveiling the heretofore mysterious realm of sulfur metabolism in LUAD and deepening our understanding of iron and copper metabolism within this context, we comprehensively compiled iron, copper, and sulfur-metabolism related genes for studying.

This study marks the inaugural comprehensive analysis of iron, copper, and sulfur metabolism in the context of LUAD. We delineate two distinct subtypes of LUAD characterized by aberrations in iron, copper, and sulfur metabolism (ICSM), and introduce an ICSM-based predictive signature, termed ICSMI, through integrated machine learning. Across multiple independent cohorts, ICSMI demonstrates significant prognostic value, surpassing the predictive power of 102 previously published LUAD prognostic models. A nomogram incorporating clinical features and ICSMI achieves commendable performance. Single-cell analyses reveal that ICSMI is most elevated in malignant cells, while cells identified as ICSMI-high phenotype via the Scissor algorithm exhibit prominent malignant attributes. Furthermore, a significant correlation is uncovered between ICSMI and TME regulators and therapeutic responsiveness, with prognostic significance extending to other cancer types. ICSMI comprises 10 ICSM-related genes (ICSMRGs), including GCDH, ST3GAL4, LDHA, FKBP4, PEBP1, DDIT4, KIF14, RRM2, SERPINB5, and ST3GAL6. Among these, ST3GAL4, LDHA, FKBP4, DDIT4, KIF14, RRM2, and SERPINB5 emerge as risk factors for LUAD, while GCDH, PEBP1, and ST3GAL6 are identified as protective factors. Previous studies have shown that LDHA can interact with APOL3 to regulate TME and ferroptosis [37, 38], meaning it’s an important TME and ferroptosis regulator. In addition, studies have shown that histone demethylated LDHA promotes lung metastasis of osteosarcoma [39], which further elucidated its risky role. In studies by Zong et al. and Meng et al., FKBP4 has been demonstrated to facilitate LUAD progression through distinct pathways, including NF-κB and mTOR [40, 41]. RRM2 was identified as a factor influencing the advancement of lung cancer and impacting the infiltration of immune cells within tumors. Inhibition of RRM2 effectively induced polarization towards M1 macrophages while suppressing M2 macrophage polarization. Additionally, treatment with the ferroptosis inhibitor ferrostatin-1 efficiently restored the balance of macrophage polarization disrupted by RRM2 inhibition [42]. Furthermore, the significant correlation between two ICSMRGs, namely KIF14 and PEBP1, and GPX4—a lipid peroxidase known for its ability to trigger ferroptosis—suggests a clear association between these genes and the ferroptotic process [43, 44]. The effects of some ICSMRGs on iron, copper, and sulfur metabolism have not been determined, but some studies suggest that they may be involved in the development of LUAD, such as SERPINB5 stimulates proliferation, metastasis, and EMT in LUAD, while elevated DDIT4 expression correlates with an unfavorable prognosis in LUAD [45, 46]. However, studies on the role of GCDH, ST3GAL4, and ST3GAL6 in LUAD and their effects on iron, copper, and sulfur metabolism are lacking. Therefore, our future research aims to explore the function of these genes in depth.

Immunotherapy presents additional chances to prolong life for LUAD patients with malignancies, thus providing a glimmer of hope for individuals grappling with this challenging illness [47]. Through the analysis of the interaction between ICSMI and the tumor microenvironment (TME), we uncovered an inverse relationship between ICSMI and the majority of immunocytes and immunomodulators. Enrichment analysis further underscored a prevalence of immunologically significant functions within low-ICSMI cohorts. Consequently, individuals exhibiting decreased ICSMI levels demonstrate "immune hot" traits characterized by intensified immunocyte infiltration. It's worth noting that prior studies have hinted at the beneficial association between heightened infiltration of most immune cells in the TME and improved prognosis for LUAD, hinting their potential role in tumor growth suppression [48]. Therefore, this observation may partly elucidate why patients with high ICSMI levels tend to have poorer prognoses. Notably, individuals with low ICSMI levels exhibited significantly lower TIDE scores, suggesting that the TIDE algorithm predicts augmented sensitivity to immunotherapy in this group. This supposition was substantiated in the GSE91061 and IMvigor210 immunotherapy cohorts, underscoring the pivotal role of ICSMI as a predictive biomarker for immunotherapeutic efficacy. Moreover, leveraging single-cell datasets, we delved into the cellular expression of ICSMI. Our findings revealed that malignant cells displayed the highest ICSMI. Subsequently, using bulk datasets, we segregated high- and low-ICSMI samples into distinct phenotypes and utilized the Scissor algorithm to project these phenotypes onto single-cell data, identifying cells closely associated with each ICSMI status. The resulting Scissor- and Scissor + phenotypes corresponded to low and high ICSMI statuses, respectively. Scissor + exhibited more aggressive features, such as reduced interaction with the TME and a significant positive correlation with poor prognosis in lung cancer patients. In contrast, Scissor- displayed enhanced interaction with the TME, bolstering immune-related functional activity and correlating with improved prognosis in lung cancer patients. Furthermore, Scissor- cells exhibited specific expression of receptors/ligands for signaling with immune cells in the TME, including CD40, TNFSF13, HLA-DRB5, and PECAM1. Notably, Scissor- exhibited higher expression levels of these molecules compared to Scissor + , and patients from the low-ICSMI group showed even higher expression in bulk data, reinforcing the concordance between Scissor ± and ICSMI-High/Low statuses and validating our conclusions.

The Oncopredict package was also used to forecast patient responsiveness to drug therapy. The findings revealed that the IC50 values of both chemotherapeutic and targeted drugs commonly employed in treating NSCLC were lower in the High-ICSMI group. This suggests that patients with lower ICSMI levels may derive greater benefits from treatment with these drugs. Additionally, in the GSE34228 dataset, we corroborated the oncopredict prediction: gefitinib-sensitive LUAD cell lines exhibited elevated ICSMI levels. Hence, immunotherapy may be a viable option for patients with low ICSMI, whereas chemotherapy and targeted therapy may be more suitable for those with high ICSMI. Moreover, in our pan-cancer analysis, we discovered that ICSMI serves as a risk factor for most cancer patients, positively associated with malignant phenotypes while inversely correlated with immune activity. This indicates that the prognostic utility of ICSMI may extend beyond LUAD patients, potentially encompassing individuals with other cancer types.

In fact, this study’s idea was inspired to some extent by Zou et al.'s research [49], which systematically incorporated genes associated with 12 different programmed cell death, and constructed a 12-gene CDI for predicting the prognosis of breast cancer patients. However, there is currently a lack of systematic studies on genes related to iron, copper, and sulfur-metabolism in cancer. In order to create a prognostic signature composed of ICSM-related genes for the first time to predict the prognosis of LUAD patients, and to provide inspiration for future research on the role of ICSM-related genes in other cancers, we designed and completed this study. After constructing the ICSMI using machine-learning methods, we found that it has higher C-Index and AUC values compared to existing clinical features such as age, gender, smoking, and clinical stage (Fig. 13A-D), and it has shown higher C-Index and HR values in almost all four independent cohorts compared to 102 previously published prognostic models. This indicates the significant value of ICSMI in predicting the prognosis of LUAD patients. Furthermore, there have been no prior studies investigating GCDH in LUAD. Therefore, our study is the first to reveal the potential role of GCDH in LUAD progression, highlighting it as a potential protective factor. Through in vitro experiments, we found that knockdown GCDH enhances the migration and invasion capabilities of two LUAD cell lines (A549 and PC9). However, the underlying mechanism behind why downregulating GCDH enhances the migration and invasion capabilities of LUAD cells remains unclear. Epithelial-Mesenchymal Transition (EMT) is a biological process during which epithelial cells undergo molecular changes that enable them to acquire mesenchymal-like characteristics. This transition involves the loss of epithelial features such as cell–cell adhesion and apical-basal polarity, accompanied by the acquisition of mesenchymal traits including increased motility, invasiveness, and resistance to apoptosis [50, 51]. EMT is a critical event in embryonic development, tissue remodeling, wound healing, and cancer progression, including lung adenocarcinoma. Therefore, we hypothesized that GCDH might promote the migration and invasion of LUAD cells through the potential mechanism of EMT. To preliminarily validate our hypothesis, we downloaded the dataset GSE114761 containing EMT information of LUAD cells and found that the expression of GCDH was significantly lower in cells undergoing EMT compared to those not undergoing EMT, and the proportion of cells undergoing EMT in the low-GCDH group is significantly higher than that in the high-GCDH groups, which suggesting that low expression of GCDH might promote EMT of LUAD cells. This is consistent with the results of our in vitro experiments (low expression of GCDH promotes migration and invasion of LUAD cells). Therefore, we speculate that EMT could be a potential mechanism by which GCDH promotes invasion and migration of LUAD cells. Although the study exhibited ICSMI's impressive potential in predicting prognosis and assessing treatment response in LUAD patients, and uncover a new protective factor—GCDH of LUAD for the first time, there exist several limitations. Firstly, the majority data employed in this research were sourced from publicly available databases. Furthermore, we did not validate the predictive efficacy of ICSMI in our own clinical cohort. The practical application of ICSMI in clinical settings requires confirmation through large-sample clinical studies. Besides, we have yet to comprehensively explore the function of specific ICSMI-associated ICSMRGs in LUAD. This study primarily relies on in vitro experiments to investigate the role of GCDH in LUAD progression. While these experiments provide valuable insights into cellular mechanisms, they fail to fully capture the complexity of tumor behavior in vivo. Therefore, the lack of in vivo validation represents a significant limitation of this study. In vivo models would allow for a more comprehensive understanding of the impact of GCDH on tumor growth, metastasis, and treatment response. Without such validation, the clinical relevance of GCDH knockout in vitro remains limit. In addition, regarding GCDH, we only investigated its correlation with migration and invasion, speculating on the potential mechanism of EMT. However, we did not validate other phenotypes possibly associated with GCDH, such as proliferation, apoptosis, etc. This is also a limitation of our study. Furthermore, we only validated the most influential ICSMRGs — GCDH’s role in the progression of LUAD. Similar validation for other ICSMRGs would further enhance the robustness of the model. Finally, a more detailed analysis of the molecular mechanisms underlying the phenotypic effects of gene expression changes would further elucidate the significance of this study. While this study has made preliminary inferences about the potential role of GCDH in influencing invasion and migration of LUAD cells through EMT, deeper mechanisms (such as which signaling pathways are affected, upstream transcription factors involved, and potential epigenetic mechanisms like methylation, phosphorylation, etc.) have not been thoroughly explored, representing further limitations of this study.

To address these limitations, future research will focus on continued follow-up and the establishment of our own cohorts to validate the performance of ICSMI in larger samples. Furthermore, we will conduct deeper investigations into GCDH, exploring its relationship with phenotypes such as proliferation and apoptosis, and committed to perform in vivo experiments to establish animal models for a more in-depth study of GCDH mechanisms in LUAD. Finally, we will employ similar methods to explore the roles of other ICSMRGs that have not been validated yet in LUAD, aiming for a more comprehensive validation of ICSMI' reliability.

In essence, our present research introduces a novel Iron, Copper, and Sulfur-Metabolism Index (ICSMI) with exceptional accuracy in forecasting the clinical trajectories of Lung Adenocarcinoma (LUAD) patients. This pioneering index not only offers profound insights into the pivotal involvement of iron, copper, and sulfur metabolisms in cancer advancement but also initiates fresh avenues for probing gene expression patterns, employing single-cell RNA-sequencing methodologies, and harnessing machine learning algorithms to refine model enhancement.

### Supplementary Information

Supplementary Material 1.

Supplementary Material 2.

## Data Availability

The datasets presented in this study can be found in online repositories. The names of the repository/repositories and accession number(s) can be found in the article/supplementary material.

## References

[1] Sung H, Ferlay J, Siegel RL, Laversanne M, Soerjomataram I, Jemal A, et al. Global Cancer Statistics 2020: Globocan Estimates of Incidence and Mortality Worldwide for 36 Cancers in 185 Countries. CA Cancer J Clin. 2021;71(3):209–49. Epub 20210204. 10.3322/caac.21660. 10.3322/caac.21660 33538338

[2] Little AG, Gay EG, Gaspar LE, Stewart AK (2007). National Survey of Non-Small Cell Lung Cancer in the United States: Epidemiology, Pathology and Patterns of Care. Lung Cancer.

[3] Chang JT, Lee YM, Huang RS (2015). The Impact of the Cancer Genome Atlas on Lung Cancer. Transl Res.

[4] Brahmer JR, Tykodi SS, Chow LQ, Hwu WJ, Topalian SL, Hwu P (2012). Safety and Activity of Anti-Pd-L1 Antibody in Patients with Advanced Cancer. N Engl J Med.

[5] Musallam KM, Taher AT (2018). Iron Deficiency Beyond Erythropoiesis: Should We Be Concerned?. Curr Med Res Opin.

[6] Hassannia B, Vandenabeele P, Vanden Berghe T (2019). Targeting Ferroptosis to Iron out Cancer. Cancer Cell.

[7] Wang S, Luo J, Zhang Z, Dong D, Shen Y, Fang Y (2018). Iron and Magnetic: New Research Direction of the Ferroptosis-Based Cancer Therapy. Am J Cancer Res.

[8] Liang C, Zhang X, Yang M, Dong X (2019). Recent Progress in Ferroptosis Inducers for Cancer Therapy. Adv Mater.

[9] Ge EJ, Bush AI, Casini A, Cobine PA, Cross JR, DeNicola GM (2022). Connecting Copper and Cancer: From Transition Metal Signalling to Metalloplasia. Nat Rev Cancer.

[10] Lelièvre P, Sancey L, Coll JL, Deniaud A, Busser B. The Multifaceted Roles of Copper in Cancer: A Trace Metal Element with Dysregulated Metabolism, but Also a Target or a Bullet for Therapy. Cancers (Basel) (2020) 12(12). Epub 20201201. doi: 10.3390/cancers12123594. 10.3390/cancers12123594 PMC7760327 33271772

[11] Denoyer D, Masaldan S, La Fontaine S, Cater MA (2015). Targeting Copper in Cancer Therapy: 'Copper That Cancer'. Metallomics.

[12] Miller CG, Schmidt EE (2020). Sulfur Metabolism under Stress. Antioxid Redox Signal.

[13] Schieber M, Chandel NS (2014). Ros Function in Redox Signaling and Oxidative Stress. Curr Biol.

[14] Liu X, Nie L, Zhang Y, Yan Y, Wang C, Colic M (2023). Actin Cytoskeleton Vulnerability to Disulfide Stress Mediates Disulfidptosis. Nat Cell Biol.

[15] Wang Z, Jensen MA, Zenklusen JC (2016). A Practical Guide to the Cancer Genome Atlas (Tcga). Methods Mol Biol.

[16] Mermel CH, Schumacher SE, Hill B, Meyerson ML, Beroukhim R, Getz G. Gistic2.0 Facilitates Sensitive and Confident Localization of the Targets of Focal Somatic Copy-Number Alteration in Human Cancers. Genome Biol (2011) 12(4):R41. Epub 20110428. doi: 10.1186/gb-2011-12-4-r41. 10.1186/gb-2011-12-4-r41 PMC3218867 21527027

[17] Clough E, Barrett T (2016). The Gene Expression Omnibus Database. Methods Mol Biol.

[18] Mariathasan S, Turley SJ, Nickles D, Castiglioni A, Yuen K, Wang Y (2018). Tgfβ Attenuates Tumour Response to Pd-L1 Blockade by Contributing to Exclusion of T Cells. Nature.

[19] Sun D, Wang J, Han Y, Dong X, Ge J, Zheng R (2021). Tisch: A Comprehensive Web Resource Enabling Interactive Single-Cell Transcriptome Visualization of Tumor Microenvironment. Nucleic Acids Res.

[20] Zhang L, Guan M, Zhang X, Yu F, Lai F (2023). Machine-Learning and Combined Analysis of Single-Cell and Bulk-Rna Sequencing Identified a Dc Gene Signature to Predict Prognosis and Immunotherapy Response for Patients with Lung Adenocarcinoma. J Cancer Res Clin Oncol.

[21] Li L, Leng W, Chen J, Li S, Lei B, Zhang H (2023). Identification of a Copper Metabolism-Related Gene Signature for Predicting Prognosis and Immune Response in Glioma. Cancer Med.

[22] Chang W, Li H, Zhong L, Zhu T, Chang Z, Ou W (2022). Development of a Copper Metabolism-Related Gene Signature in Lung Adenocarcinoma. Front Immunol.

[23] Zhao M, Li M, Zheng Y, Hu Z, Liang J, Bi G (2022). Identification and Analysis of a Prognostic Ferroptosis and Iron-Metabolism Signature for Esophageal Squamous Cell Carcinoma. J Cancer.

[24] Yao J, Chen X, Liu X, Li R, Zhou X, Qu Y (2021). Characterization of a Ferroptosis and Iron-Metabolism Related Lncrna Signature in Lung Adenocarcinoma. Cancer Cell Int.

[25] Liberzon A, Birger C, Thorvaldsdóttir H, Ghandi M, Mesirov JP, Tamayo P (2015). The Molecular Signatures Database (Msigdb) Hallmark Gene Set Collection. Cell Syst.

[26] Jiang P, Gu S, Pan D, Fu J, Sahu A, Hu X (2018). Signatures of T Cell Dysfunction and Exclusion Predict Cancer Immunotherapy Response. Nat Med.

[27] Wilkerson MD, Hayes DN (2010). Consensusclusterplus: A Class Discovery Tool with Confidence Assessments and Item Tracking. Bioinformatics.

[28] Yu G, Wang LG, Han Y, He QY (2012). Clusterprofiler: An R Package for Comparing Biological Themes among Gene Clusters. Omics.

[29] Hänzelmann S, Castelo R, Guinney J (2013). Gsva: Gene Set Variation Analysis for Microarray and Rna-Seq Data. BMC Bioinformatics.

[30] Zeng D, Ye Z, Shen R, Yu G, Wu J, Xiong Y (2021). Iobr: Multi-Omics Immuno-Oncology Biological Research to Decode Tumor Microenvironment and Signatures. Front Immunol.

[31] Sun D, Guan X, Moran AE, Wu LY, Qian DZ, Schedin P (2022). Identifying Phenotype-Associated Subpopulations by Integrating Bulk and Single-Cell Sequencing Data. Nat Biotechnol.

[32] Fang Z, Tian Y, Sui C, Guo Y, Hu X, Lai Y (2022). Single-Cell Transcriptomics of Proliferative Phase Endometrium: Systems Analysis of Cell-Cell Communication Network Using Cellchat. Front Cell Dev Biol.

[33] Yang W, Soares J, Greninger P, Edelman EJ, Lightfoot H, Forbes S, et al. Genomics of Drug Sensitivity in Cancer (Gdsc): A Resource for Therapeutic Biomarker Discovery in Cancer Cells. Nucleic Acids Res (2013) 41(Database issue):D955–61. Epub 20121123. doi: 10.1093/nar/gks1111. 10.1093/nar/gks1111 PMC3531057 23180760

[34] Maeser D, Gruener RF, Huang RS. Oncopredict: An R Package for Predicting in Vivo or Cancer Patient Drug Response and Biomarkers from Cell Line Screening Data. Brief Bioinform (2021) 22(6). doi: 10.1093/bib/bbab260. 10.1093/bib/bbab260 PMC8574972 34260682

[35] Van de Sande B, Flerin C, Davie K, De Waegeneer M, Hulselmans G, Aibar S (2020). A Scalable Scenic Workflow for Single-Cell Gene Regulatory Network Analysis. Nat Protoc.

[36] Qin J, Xu Z, Deng K, Qin F, Wei J, Yuan L (2021). Development of a Gene Signature Associated with Iron Metabolism in Lung Adenocarcinoma. Bioengineered.

[37] Lv Y, Tang W, Xu Y, Chang W, Zhang Z, Lin Q (2023). Apolipoprotein L3 Enhances Cd8+ T Cell Antitumor Immunity of Colorectal Cancer by Promoting Ldha-Mediated Ferroptosis. Int J Biol Sci.

[38] Feng Y, Dai Y (2023). Apol3-Ldha Axis Related Immunity Activation and Cancer Ferroptosis Induction. Int J Biol Sci.

[39] Jiang Y, Li F, Gao B, Ma M, Chen M, Wu Y (2021). Kdm6b-Mediated Histone Demethylation of Ldha Promotes Lung Metastasis of Osteosarcoma. Theranostics.

[40] Zong S, Jiao Y, Liu X, Mu W, Yuan X, Qu Y (2021). Fkbp4 Integrates Fkbp4/Hsp90/Ikk with Fkbp4/Hsp70/Rela Complex to Promote Lung Adenocarcinoma Progression Via Ikk/Nf-Κb Signaling. Cell Death Dis.

[41] Meng W, Meng J, Jiang H, Feng X, Wei D, Ding Q (2020). Fkbp4 Accelerates Malignant Progression of Non-Small-Cell Lung Cancer by Activating the Akt/Mtor Signaling Pathway. Anal Cell Pathol (Amst).

[42] Tang B, Xu W, Wang Y, Zhu J, Wang H, Tu J (2021). Identification of Critical Ferroptosis Regulators in Lung Adenocarcinoma That Rrm2 Facilitates Tumor Immune Infiltration by Inhibiting Ferroptotic Death. Clin Immunol.

[43] Jiao H, Yang H, Yan Z, Chen J, Xu M, Jiang Y (2021). Traditional Chinese Formula Xiaoyaosan Alleviates Depressive-Like Behavior in Cums Mice by Regulating Pebp1-Gpx4-Mediated Ferroptosis in the Hippocampus. Neuropsychiatr Dis Treat.

[44] Feng Z, Li B, Chen Q, Zhang H, Guo Z, Qin J (2022). Identification and Validation of a Gpx4-Related Immune Prognostic Signature for Lung Adenocarcinoma. J Oncol.

[45] He X, Ma Y, Huang Z, Wang G, Wang W, Zhang R (2023). Serpinb5 Is a Prognostic Biomarker and Promotes Proliferation, Metastasis and Epithelial-Mesenchymal Transition (Emt) in Lung Adenocarcinoma. Thorac Cancer.

[46] Song L, Chen Z, Zhang M, Zhang M, Lu X, Li C (2021). Ddit4 Overexpression Associates with Poor Prognosis in Lung Adenocarcinoma. J Cancer.

[47] Ruiz-Cordero R, Devine WP (2020). Targeted Therapy and Checkpoint Immunotherapy in Lung Cancer. Surg Pathol Clin.

[48] Chen H, Lin R, Lin W, Chen Q, Ye D, Li J (2022). An Immune Gene Signature to Predict Prognosis and Immunotherapeutic Response in Lung Adenocarcinoma. Sci Rep.

[49] Zou Y, Xie J, Zheng S, Liu W, Tang Y, Tian W (2022). Leveraging Diverse Cell-Death Patterns to Predict the Prognosis and Drug Sensitivity of Triple-Negative Breast Cancer Patients after Surgery. Int J Surg.

[50] Lamouille S, Xu J, Derynck R (2014). Molecular Mechanisms of Epithelial-Mesenchymal Transition. Nat Rev Mol Cell Biol.

[51] Dongre A, Weinberg RA (2019). New Insights into the Mechanisms of Epithelial-Mesenchymal Transition and Implications for Cancer. Nat Rev Mol Cell Biol.

