# Supplementary material for: Machine-learning developed an iron, copper, and sulfur-metabolism associated signature predicts lung adenocarcinoma prognosis and therapy response
Source: Respir Res. 2024 May 14;25:206. doi: 10.1186/s12931-024-02839-6 (PMC11092068; doi:10.1186/s12931-024-02839-6)
Supplement: Supplementary file 2 — Supplementary Material 2. [file 12931_2024_2839_MOESM2_ESM.docx]

**Supplementary methods**

**Generation of ICSMI**

To establish a robust and accurate consensus model (ICSMI), we employed a comprehensive ensemble of 10 distinct machine learning algorithms. These included the random survival forest (RSF), elastic network (Enet), Lasso, Ridge, stepwise Cox, CoxBoost, partial least squares regression for Cox (plsRcox), supervised principal components (SuperPC), generalized boosted regression modeling (GBM), and survival support vector machine (survival-SVM).

Several algorithms demonstrated adeptness in feature selection, including Lasso, stepwise Cox, CoxBoost, and RSF. To create a consolidated model, these algorithms were amalgamated. The amalgamation process involved executing 114 unique algorithm combinations, each tailored to form prediction models within the leave-one-out cross-validation (LOOCV) framework.

The inaugural discovery of the signature was performed utilizing TCGA-LUAD data. For the RSF model, we employed the randomForestSRC package, configuring its ntree and mtry parameters through grid-search within the LOOCV framework. The optimal parameter pair (ntree, mtry) resulting in the highest C-index value was selected.

Enet, Lasso, and Ridge implementations utilized the glmnet package, determining the regularization parameter λ via LOOCV, while α, the L1-L2 trade-off parameter, was systematically varied from 0 to 1 in intervals of 0.1.

Implementing the stepwise Cox model through the survival package involved applying a stepwise algorithm based on the Akaike information criterion (AIC). We experimented with "both", "backward", and "forward" directions for stepwise search.

The CoxBoost model was realized using the CoxBoost package, with optimal penalty determined by LOOCV's optimCoxBoostPenalty function. Subsequently, the number of boosting steps was selected via cv.CoxBoost, and the multivariate Cox model's dimension was established by the main CoxBoost routine.

For the plsRcox model, the plsRcox package was utilized. We determined the necessary number of components using the cv.plsRcox function, fitting a partial least squares regression generalized linear model with the plsRcox function.

The SuperPC model, sourced from the superpc package, extended principal component analysis to extract prominent variation directions within a dataset.

The GBM model was executed through the gbm package. Employing the LOOCV technique, the cv.gbm function efficiently determined the index for the number of trees, aiming to minimize the cross-validation error. Subsequently, the gbm function was applied to effectively configure the generalized boosted regression model.

Additionally, the survival-SVM model was established utilizing the survivalsvm package. This model employed a regression approach that thoughtfully incorporated the consideration of censoring. This integration ensured the formulation of inequality constraints for the support vector problem adequately accounted for the specific challenges posed by censoring.

**Supplementary Figures**


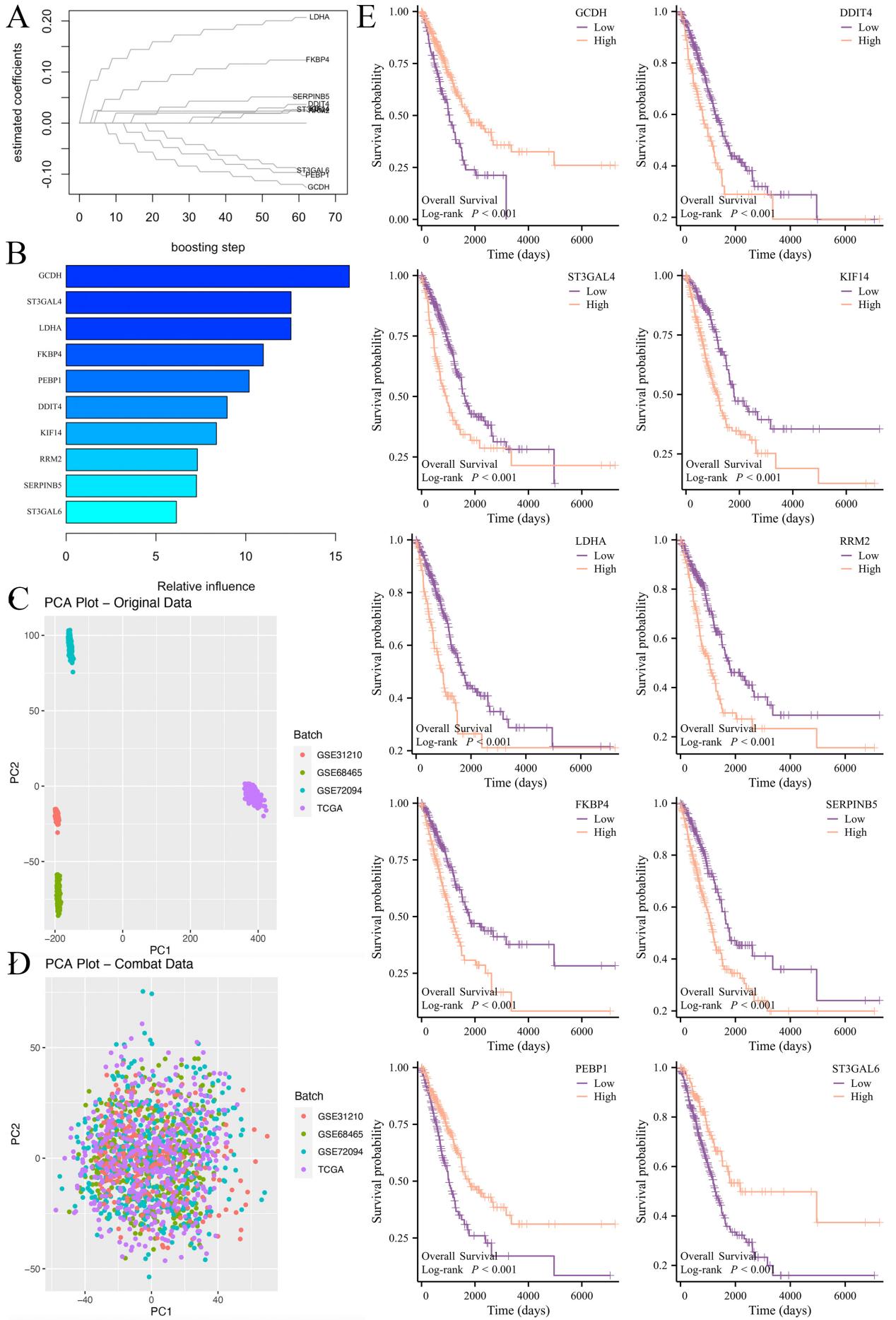


**Supplementary Figure 1.** (A) CoxBoost identified ten hub ICSMRGs. (B) Ten ICSMGRs’ relative influence on ICSMI. (C) PCA plot shows that distinct batch effect exists in four datasets. (D) PCA plot shows that the batch effect has been removed well. (E) Ten ICSMRGs’ influence on LUAD patients’ survival.
